# Brain volume increase and neuronal plasticity underly predator-induced morphological defense expression in *Daphnia longicephala*

**DOI:** 10.1038/s41598-021-92052-y

**Published:** 2021-06-15

**Authors:** A Graeve, I Ioannidou, J Reinhard, D. M. Görl, A Faissner, LC Weiss

**Affiliations:** 1 Department of Animal Ecology, Evolution and Biodiversity, Ruhr-University Bochum, Universitätsstrasse 150, 44780 Bochum, Germany; 2 Department of Cell Morphology and Molecular Neurobiology, Ruhr-University Bochum, Universitätsstrasse 150, 44780 Bochum, Germany

**Keywords:** Synaptic plasticity, Ecophysiology, Evolutionary ecology

## Abstract

Predator-induced phenotypic plasticity describes the ability of prey to respond to an increased predation risk by developing adaptive phenotypes. Upon the perception of chemical predator cues, the freshwater crustacean *Daphnia longicephala* develops defensive crests against its predator *Notonecta* spec. (Heteroptera). Chemical predator perception initiates a cascade of biological reactions that leads to the development of these morphological features. Neuronal signaling is a central component in this series, however how the nervous system perceives and integrates environmental signals is not well understood. As neuronal activity is often accompanied by functional and structural plasticity of the nervous system, we hypothesized that predator perception is associated with structural and functional changes of nervous tissues. We observe structural plasticity as a volume increase of the central brain, which is independent of the total number of brain cells. In addition, we find functional plasticity in form of an increased number of inhibitory post-synaptic sites during the initial stage of defense development. Our results indicate a structural rewiring of nerve-cell connections upon predator perception and provide important insights into how the nervous system of prey species interprets predator cues and develops cost–benefit optimized defenses.

## Introduction

**Figure 1:**
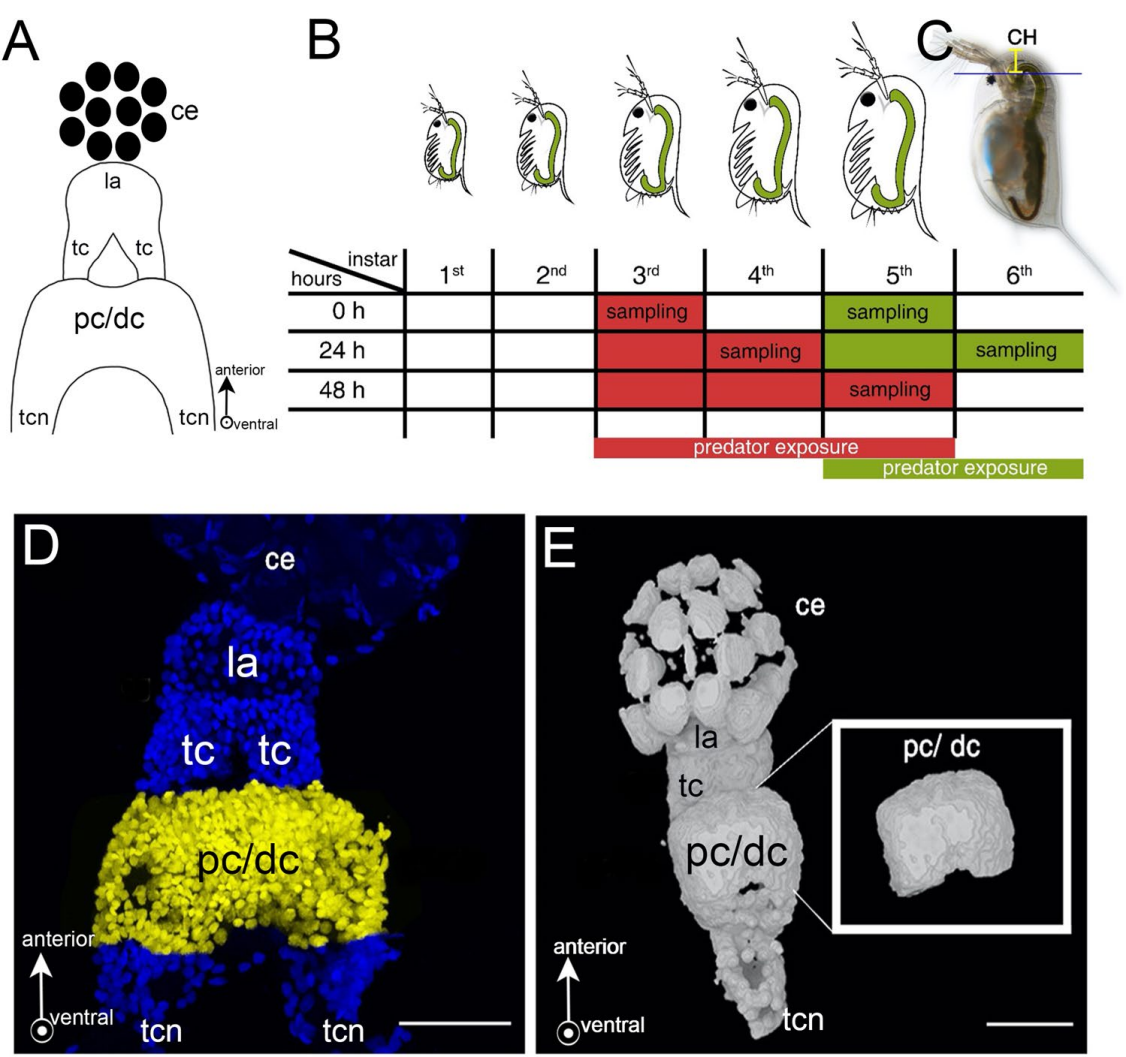
Schematic overview of experimental procedures. (**A﻿**) Schematic frontal view of a *Daphnia* brain displaying the optic ganglia: composed of lamina and tecta, and pc/dc-complex. (**B**) Points in time of *D. longicephala* induction. Predator exposure started in the 3rd or 5th instar, respectively. When exposed in the 3rd instar, individuals were sampled at 0 h, 24 h and 48 h. To validate our findings in a different instar, animals were also exposed in the 5th instar; here we focussed on the target stages i.e. 0 h and 24 h. Controls were performed alongside the respective treatment but without predators. (**﻿C**) Measurement of morphological defence parameters. We measured the crest height as the distance between the upper eye margin and the rostral extension of the head (yellow line). (**D**) Dissected brain mount stained with DAPI. We counted the cells of the pc/dc-complex (yellow). (**E**) 3D projection of a brain mount from confocal stacks for volumetrics created with MorphoGraphX Version 1.1.1280-CellAtlas (available from https://www.mpipz.mpg.de/MorphoGraphX/software). Only the pc/dc-complex (insert) was used to determine the brain volumes. (**﻿A**, **D**, **E**) Arrows depict the anterior and ventral (arrow points out of the drawing pane) orientation. Abbreviations: CH, crest height; ce, compound eye; la, lamina; tc: tecta, pc/dc, pc/dc-complex; tcn, tritocerbral neuropil.

Phenotypic plasticity describes the ability of an organism with a given genotype to adapt its phenotype in response to changing environmental conditions^1^. One well-known example of phenotypic plasticity is inducible defenses. Here, the presence of predators, indicated by chemical cues (kairomones), induces defensive features in the prey, which make them less susceptible to predation. The freshwater crustacean *Daphnia* is a prime example of predator-induced defenses^2^, which range from morphological features such as helmets in *D. cucullata*^3^, neck teeth in *D. pulex*^4^ or crests in *D. longicephala*^5^ to changes in life history^6,7^ or adaptive behavior^8^. Interestingly, morphological defenses are not only expressed in a predator-density specific manner, but rather they are fine-tuned to the predation risk, so that also the conspecific density is decisional for the degree of defense expression^9^. Such condition-dependent adjustments require a nervous system capable to decipher different chemical cues from the heterogeneity of many environmental factors and transfer this into a cost–benefit optimized phenotype. *Daphnia* perceive chemical cues via the antennules, upon which a signaling cascade involving cholinergic, dopaminergic, glutamatergic and GABAergic components is initiated, which ultimately leads to the transformation of the undefended into the defended phenotype^10–15^. It is still unknown how the nervous system detects and integrates environmental information when the *Daphnia* brain morphologically appears rather simple (Fig. 1A). It is subdivided into three main neuropils: protocerebrum, deutocerebrum and tritocerebrum^16^. The protocerebrum receives direct visual input from the compound eye via the optic ganglia (lamina and tecta) and chemosensory input from the antennules via the deutocerebrum^16^. Via the circumoral connectives, the protocerebrum is connected to the tritocerebrum. This consists of two ganglia inferior to the esophagus that are transversally connected by the tritocerebral commissures. These two neuropils connect with the mandibular ganglia and extend into the rope-ladder-like nerve cord innervating the body and appendages^16^. Between these three neuropils, the protocerebrum comprises the largest part of the brain and is discussed to be centrally involved in the analysis and interpretation of environmental sensory signals^17^. Such environmental signal integration is often associated with changes in form and function of nervous systems. While changes in form are referred to as structural brain plasticity, changes in function are referred to as functional plasticity^18^. Structural plasticity has been reported in vertebrates and invertebrates, e.g. rats living in complex environments show larger brains with larger cells and more dendritic fields than rats raised in homogeneous, uncomplex environments^19^. Tadpoles of the leopard frog develop smaller and narrower brains upon an emerging predator threat^20^. Similarly, different insect species have been found to undergo structural brain plasticity associated with learning, food search and novel tasks^21–24^. In honeybees certain brain regions, i.e. the mushroom bodies in the protocerebrum, experience anatomical reorganization associated with labor division due to differences in foraging experience^25^. *Drosophila* develop larger brain structures in diversified environments than in isolation^26^. All these structural changes are discussed to be cost–benefit optimized so that the neuronal structures become as large as needed in order to be highly effective for the given task. At the same time brain size is limited as nervous tissues are energetically demanding^27^. In contrast to structural brain plasticity, functional brain plasticity affects the nervous system´s function by altering the abundance and reactive strength of neuronal connections^28^. Neuronal connections are e.g. made at chemical synapses. These consist of a presynaptic bouton with the active zone (AZ), responsible for the regulated release of neurotransmitters, and the postsynaptic density (PSD), representing the neurotransmitter reception apparatus. These synaptic sites are intermitted by the synaptic cleft into which neurotransmitters are released. Neuronal activity can either trigger neurotransmitter release at excitatory synapses, increasing the activity of the postsynaptic neuron, or at inhibitory synapses, reducing neuronal activity^29^. Functional plasticity by means of differences in postsynaptic receptor abundance has been shown in the avian brain during the filial imprinting process^30^ and in mammals experiencing enriched environments^19,31^. As space is the factor limiting the number of synaptic connections in one neuron^32^, only an enlargement of the neuronal surface area can increase synaptic connections. The surface area can increase through an enlarged cell volume and when many cells enlarge their volume, the overall neuropil mass will increase and is therefore a component of structural plasticity^18^. Thus, structural and functional brain plasticity are closely related as the formation of new neuronal connections requires space^19^ and may therefore be able to shape neuropil form and size. Especially inhibitory synapses are important with regard to the control and regulation of overall neuronal excitability within neuronal networks of cognitive processes^33,34^. The scaffolding protein gephyrin is a central component of the inhibitory nervous system, as it anchors GABA_A_ and glycine receptors at the PSD^35^. In addition, this protein is involved in post-Golgi transport of the respective receptors to the PSD^36^. Taken together, gephyrin is a good indicator of functional plasticity, as it indicates activity changes of the inhibitory PSD by receptor shuffling.

Here, we investigated structural and functional brain plasticity underlying predator perception in *Daphnia longicephala.* As cognition is a central task of the protocerebrum^37^, we focused on the proto- and deutocerebrum (pc/dc-complex), which are indistinct in *Daphnia*. We determined pc/dc-complex volume together with a reorganization of neuronal circuitry by determination of the number of neuronal cells. To exclude that alterations in pc/dc-complex volume are a result of the predator induced overall body growth and the crest, we also determined the volume of the optic ganglia (OG). Additionally, we investigated cells expressing the anchoring protein gephyrin in pc/dc-complexes of predator-exposed and control *D. longicephala*. In order to exclude an age-dependent effect, we conducted the experiment at two different developmental points in time. We demonstrate that there is structural and functional plasticity independent of developmental age associated with predator perception.

## Material and methods

### *Daphnia longicephala* culture

*D. longicephala* clone LP1 (Lara-Pond, Australia) was cultured in artificial *Daphnia* medium (ADaM (Klüttgen et al. 1994)) in 1 L beakers (Weck®, Germany) containing 20–25 age-synchronized individuals under constant day:night conditions (16 h:8 h) at 20 ± 1 °C. Animals were fed ad libitum with the algae *Acutodesmus obliquus*. The beakers were cleaned every 48 h. Half of the medium was exchanged weekly.

### Predator culture

Backswimmers (*Notonecta* spec.) were collected from the ponds of the Ruhr-University Botanical Gardens. Animals were maintained in 1 L beakers (WECK®, Germany) filled with charcoal-filtered tap water under standardized conditions (20 ± 1 °C with 16 h:8 h light:dark cycle). They were fed regularly ad libitum with *Daphnia* spec.

### Experimental conditions

All animals of the culture and the experiments were maintained in ADaM and reared at 20 °C ± 0.1 °C in incubators at constant light with a 16 h:8 h day:night cycle.

### Experimental setup

We collected 14 h (± 1 h) old *D.* *longicephala* from age-synchronized cultures and reared 30 animals per 1 L beaker. Once the animals reached the 3rd instar i.e., after the successful completion of two molting cycles, they were transferred into predator exposure or control conditions. For the predator exposure, net cages (mesh size 100 µm) containing one *Notonecta* spec. were placed into 1 L beakers. Notonectids were fed with five *D.* *longicephala* per day to ensure continuous kairomone exposure. The experimental *D. longicephala* were located outside of the net cage, which prevented predators from feeding on the test specimens. Predator exposure was conducted for 48 h until *D. longicephala* reached the 5th instar. Control treatments were cultivated accordingly, but without predators. Control and predator-exposed specimens were collected at explicit points in time and treatment from the moment of predator exposure i.e. 0 h, 24 h, and 48 h. Per point in time and treatment, we collected 20 to 30 *D.* *longicephala* (Fig. 1B, red). The complete experimental set-up was repeated 9 times. Since *D. longicephala* is responsive to predator cues also at later stages^15^, we also conducted experiments where predator exposure started in the 5th instar (Fig. 1B, green) focusing on the relevant points in time i.e. 0 h and 24 h and replicated this 3 times.

### Measurement of defensive features

We measured defense expression at above-described points in time using a SZX16 stereomicroscope (Olympus, Japan) equipped with a TSO digital-camera (TSO-KST 6000849) controlled by the software Vidmess Version 3.0 (TSO Thalheim Spezialoptik GmbH, Germany). We measured the crest height from the upper margin of the compound eye to the distal height of the crest (Fig. 1C).

### Immunohistochemistry

Animals were fixed using 4% PFA (formaldehyde (J.T. Baker) diluted in phosphate-buffered saline (PBS) 0.1 M; pH 7.4) for 5 min. Then, we dissected the pc/dc-complex, still attached to the optic ganglia and compound eye (prospectively referred to as ‘brain mounts’) using fine forceps. We used the dark pigmented compound eye as a visual guide to facilitate working with the small and unpigmented neuronal tissue. Brain mounts were transferred to poly-L-lysine coated object slides (VWR, Germany). All subsequent incubation steps were performed in a humidified chamber and on an orbital shaker (neoLab®, Germany) at 15 rpm and 4 °C. Brain mounts were post-fixed for 10 min in 4% PFA and rinsed with PBS 3*5 min. Fixed brain mounts were blocked with 5% goat normal serum (Dianova, Germany) diluted in PBS-TX 0.1% (PBS, 0.1 M; pH 7.4 with 0.05% Triton-X 100; Sigma Aldrich, Germany) for 3 h. Subsequently, we applied the primary antibody anti-gephyrin raised in mouse (Cat. No. 147011; Synaptic Systems, Germany; RRID:AB_887717; 1:100 in PBS-TX 0.1%). Samples were incubated overnight and then washed with PBS-TX 0.1% 3*10 min. The secondary antibody anti-mouse Alexa^488^ (Dianova, Germany; RRID:AB_2338845) was applied (1:250 diluted in PBS-TX 0.1%) and incubated for 2 h. Brain mounts were rinsed 3*10 min with PBS-TX 0.1%, mounted in DAPI-Vectashield (Vector Laboratories, USA) for nuclei staining and preservation. Slides treated with secondary antibody only and DAPI-Vectashield served as negative controls (Fig. S1A, B). In order to confirm that gephyrin is associated with inhibitory glycinergic neurons in *Daphnia*, we performed a double staining using the anti-gephyrin antibody and an anti-glycine receptor (GlyR) antibody raised in rabbit (Cat. No. 146008; Synaptic Systems, Germany; RRID:AB_2636914) (1:50 diluted in PBS-TX 0.1%). This antibody binds to the glycine receptor α1-subunit. Detection was performed with the secondary antibody anti-rabbit^594^ (1:250) (Dianova, Germany; RRID:AB_2307325) on brain mounts of adult *D. longicephala*. Every step was performed as described above. Antibody validation is described in the supplement (Fig. S2).

### Confocal images

We acquired image stacks of the *D. longicephala* brain mounts using a confocal laser scanning microscope (SP5II, Leica Microsystems, Wetzlar, Germany) and Leica cLSM (SP8, Leica Microsystems, Germany). Image stacks were acquired with a 40× water immersion objective, a 63× and a 100× oil immersion objective. Step intervals were chosen between 0.5 and 0.8 µm, according to nuclear size ensuring the recording of all cells in one stack. Figures were composed and labelled in Photoshop CS 4 (Adobe Systems, San Jose, CA). Brightness, saturation, and contrast were optimized; no further image editing was conducted.

### Cell counting and volumetric measurement in pc/dc-complexes and OG

3D projections of captured image stacks were composed in ImageJ 1.52n (Fiji)^38^. The total number of nerve cells (identified with the nuclear marker 2-[4-(Aminoiminomethyl)phenyl]-1H-Indole-6-carboximidamide hydrochloride (DAPI) and the number of gephyrin-labelled cells in the pc/dc-complex were counted manually in 3D projections using the ‘multipoint tool’ (Fig. 1D). To determine the pc/dc-complex and OG volume, we used cLSM image stacks of the brain mount DAPI staining and created tif-stacks with Fiji for MorphoGraphX import^39^ (Fig. 1E). Here, we used the ´Gaussian Blur` with a rate of 1 µm in each direction. Using ´Marching Cubes Surface` we created a mesh surface with a cube size of 1 µm. Since we aimed to measure only the pc/dc-complex or OG volume respectively, we removed the surrounding parts of the brain mount using the ´pixel edit` tool (Fig. 1E insert). Each stack was saved as ply-file before conversion into stl-files using MeshLab^40^. These files were required for the analysis in Blender, an open-source 3D graphics and animation package. The stl-files were opened in Blender and the volume was calculated with the ‘3D printing’ and ‘volume’ function as described in Horstmann et al.^41^ (Fig. 1E). OG volume was only measured in individuals that were exposed from the 3rd to the 5th instar.

### Statistical analyses

We tested for normal distribution using a Shapiro–Wilk test. Only crest height, and brain volume followed a normal distribution, so that all other data was log-transformed. To analyze crest expression in line with structural and functional plasticity across three instars starting from the third to the fifth instar we used a one-way analysis of variance (ANOVA) with instar as factor. By this we determined age-dependence of the measured parameters (i.e. crest height, brain volume, OG volume, log(#gephyrin-labelled cells), log(#DAPI-labelled cells), log(#gephyrin/#DAPI-labelled cells) individually per treatment (control; induced), as the interaction of stage and instar was irrelevant to our hypothesis. Post hoc comparisons to determine differences between individual instars were performed using a Tukey Test. We then determined differences between control and predator-induced groups using a Student’s t-test for each developmental instar and adjusted the significance threshold for multiple testing using the alpha-correction. The analysis of the control experiment using a later instar only comprised two instars, so that we investigated for differences between instar and treatment using a Student’s t-test. Levels of significance were again adjusted for multiple testing.

Data is displayed untransformed. Statistics were performed in STATISTICA 14 (Statsoft inc.) plots were made in R using the ggplot2 package^42,43^.

## Results

### Inducible defenses

**Figure 2:**
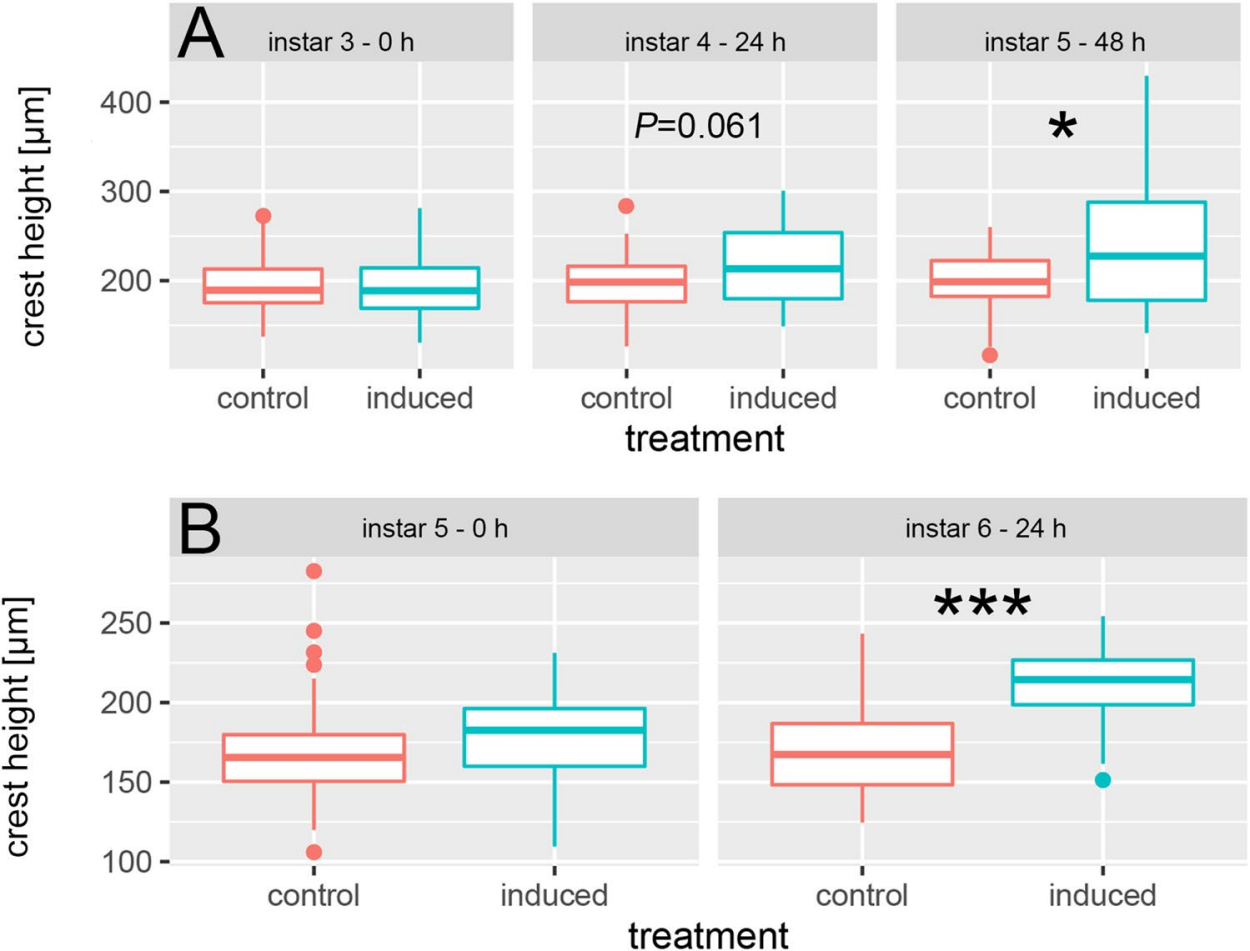
Morphological defense expression *in D. longicephala* exposed to predators in the 3rd instar. (**A**) Parameters were directly determined at 0 h (3rd instar), and subsequently monitored after 24 h (4th instar), and 48 h (5th instar). Crest height shows a tendency towards an increase in predator-exposed *D.* *longicephala* after 24 h (*P* = 0.061; t =  − 1.91; df = 57). After 48 h predator-exposed individuals have a significantly increased crest height compared to controls (*P* = 0.01; t =  − 2.64; df = 68). Alpha corrected levels of significance: *P ≤ 0.017; ***P ≤ 0.0003. (**B**) Predator exposure started in the 5th instar and parameters were directly determined at 0 h (5th instar), and subsequently after 24 h (6th instar). Crest height is significantly increased after 24 h predator exposure (*P* ≤ 0.001; t = −4.18; df = 44). Alpha corrected levels of significance: ***P ≤ 0.0005; Table S1.

Crests are constant across instars in the control group but grow significantly larger in the predator-induced treatment. Across instars, defenses grow significantly, so that we find a significantly increased crest height in predator-exposed animals after 48 h (5th instar) compared to 0 h (3rd instar, Fig. 2A; Tables S1, S2). Within this time, crests increased 1.2-fold in the predator exposed treatment and remain constant in size in the control treatment (1.0×).

We find a tendency towards an increased crest expression in predator-exposed animals compared to controls after 24 h (4th instar; (*P* = 0.061; t =  − 1.91; df = 57) and a significantly increased crest height after 48 h (5th instar; *P* = 0.01; t =  − 2.64; df = 28, Table S3). Individuals that were exposed to predator cues in the 5th instar also showed an increased crest expression after 24 h predator exposure (*P* ≤ 0.001; t = 4.18; df = 44; Fig. 2B; Tables S4, S5).

### Structural plasticity in the pc/dc-complex

#### Volumetric changes of the pc/dc-complex

**Figure 3:**
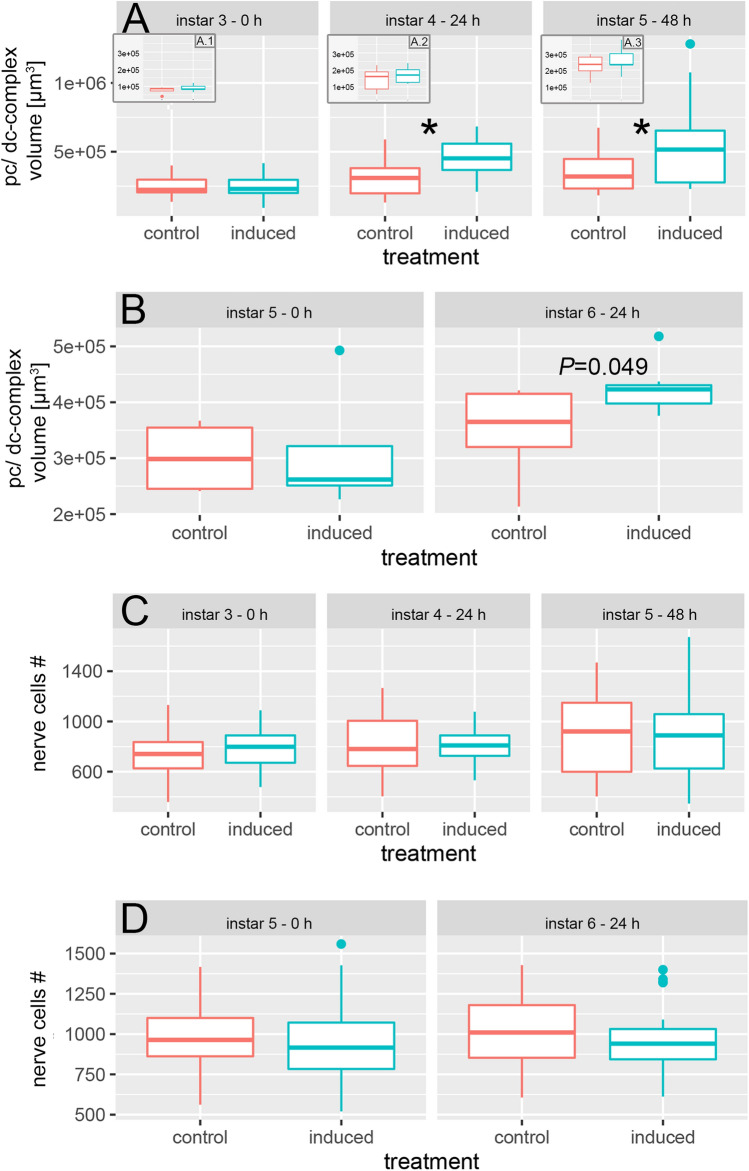
Structural plasticity underlying morphological defense expression (**A**) 24 h predator exposure starting from the 3rd instar, significantly induced an increase in pc/dc-complex volume compared to the controls (*P* = 0.011; t = 2.73; df = 28). This increase was also observed after 48 h predator-exposure (*P* = 0.014; t =  − 2.59; df = 33). **(A**.**1**, **A**.**2**, **A**.**3)**. We detected no significant differences in OG volume between treatments. We observed a significant increase in OG volume between instar 3 and instar 5 in both treatments (control ANOVA: F_(2;13)_ = 9.63 *P* = 0.003 Tukey: *P* = 0.002; induced ANOVA: F_(2;14)_ = 12.07 *P* = 0.001 Tukey: *P* ≤ 0.001). Alpha corrected levels of significance: * *P* ≤ 0.017; (**B**) When exposed to predators from the 5th instar onwards, the pc/dc-complex volume shows a tendency towards an increase after 24 h predator exposure compared to the control (*P* = 0.049; t =  − 2.19; df = 12) and to the 5th instar (*P* = 0.036; t =  − 2.42; df = 10) Alpha corrected levels of significance: *P ≤ 0.025. (**C**) We detected no significant differences in nerve cell numbers between treatments. We found a tendency towards an increased nerve cell number in naïve individuals in the 5th instar compared to the 3rd instar, when the animals were exposed to predators from the 3rd instar onwards (ANOVA: F_(2;98)_ = 2.64 *P* = 0.077 Tukey: *P* = 0.076) Alpha corrected levels of significance: * *P* ≤ 0.025. (**D**) When exposed to predators from the 5th instar onwards, we find no differences in nerve cell numbers between treatments or instars. Alpha corrected levels of significance: *P ≤ 0.017.

We observed increasing pc/dc-complex volumes in both treatments with age. Naïve, control individuals showed a mean volume of 249,348.70 µm^3^ ± 70,955.12 in the 3rd instar and a mean volume of 353,098.40 ± 142,285.70 µm^3^ in the 5th instar (Fig. 3A, Tables S1, S2). In predator-exposed animals pc/dc-complex volume increased significantly from 247,623.70 µm^3^ ± 97,067.05 µm^3^ in the 3rd instar to 451,761.40 µm^3^ ± 146,165.9 µm^3^ in the 4th instar and to 565,640.5 µm^3^ ± 340,501.1 µm^3^ in the 5th instar (Fig. 3A; Tables S1, S2). We did not observe volumetric changes in pc/dc-complexes of naïve individuals from the 5th to the 6th instar (Fig. 3B, Table S4). Predator-exposed *D. longicephala* showed an increase in pc/dc-volumes after 24 h (mean 5th instar: 310,848.2 µm^3^ ± 122,657 µm^3^; mean 6th instar: 424,134.2 µm^3^ ± 43,543.65 µm^3^; Fig. 3B, Table S5). When animals were exposed to the predator in the 3rd instar, no significant difference between control and predator-exposed *D. longicephala* pc/dc-complex volumes was observed at 0 h (Table S3). Twenty-four hours later, pc/dc-complex volume in predator-exposed individuals was significantly larger than in 24 h controls with a mean of 451,761.40 ± 146,165.90 µm^3^ (Fig. 3A, Table S3). In 48 h predator-exposed *D. longicephala*, the volume was also significantly increased compared to the control (mean induced: 565,640.50 µm^3^ ± 340,501.1; Fig. 3A, Table S3). During this time the pc/dc volume increases 1.4-fold in control animals, and 2.2-fold in predator exposed animals. Accordingly, among individuals exposed in the 5th instar, we detected a significant increase in pc/dc-complex volumes of predator-exposed animals compared to the control after 24 h (mean control: 350,290.4 µm^3^ ± 81,781.9 µm^3^, mean induced: 424,134.0 µm^3^ ± 43,543.70; Fig. 3B; Tables S4, S5).

#### Volumetric changes of the OG

Control and predator-exposed *D. longicephala* show an increase in OG volume with increasing age so that animals in the 5th instar have significantly larger OG than animals in the 3rd instar (mean control 3rd instar: 75,837.76 µm^3^ ± 21,080.22 µm^3^, mean control 5th instar: 226,191.2 µm^3^ ± 63,729.89 µm^3^; mean induced 3rd instar: 89,783.36 µm^3^ ± 20,623.36 µm^3^, mean induced 5th instar: 259,253.9 µm^3^ ± 81,789.8 µm^3^; Fig. 3A; Tables S1, S2). We observed no significant differences in OG volume between control and predator-exposed individuals at any point in time (Fig. 3A; Tables S3, S4, S5). Optic ganglia of control specimens increase by 2.9-fold and of predator induced specimens by 2.88-fold over the developmental stages.

#### Nerve cell numbers in the pc/dc-complex

We detected a tendency towards an increased number of cells in the pc/dc-complex of naïve *D.* *longicephala* from 734.86 ± 180.13 cells in the 3rd to 903.18 ± 319.73 cells in the 5th instar (Fig. 3C; Tables S1, S2). Across developmental stages the number of cells increase 1.23-fold irrespective of the treatment. Between control and predator-exposed specimens, we did not detect differences in nerve cell numbers at any point in time (Fig. 3C, D; Tables S3, S4).

### Functional plasticity in the pc/dc-complex

#### Characterization of synaptic composition

**Figure 4:**
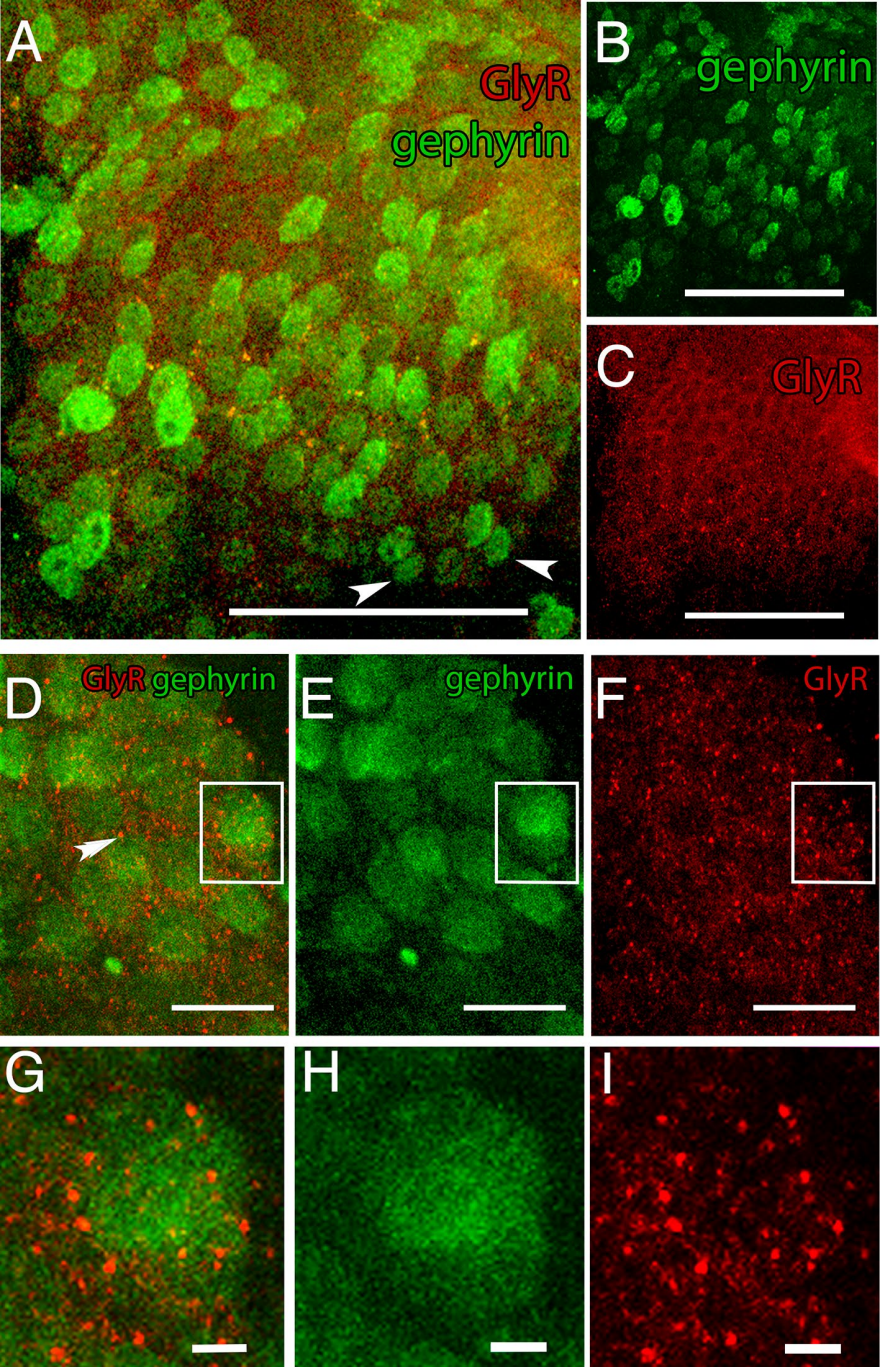
Co-localization of gephyrin and glycine receptor (GlyR). (**A**) Merged display of anti-gephyrin and anti-GlyR staining pattern. Both antibodies are identified within the pc/dc-complex. Anti-GlyR staining colocalized with the anti-gephyrin antibody staining. Not all gephyrin-labelled cells show markings of anti-GlyR (white arrow heads). (**B**) Gephyrin is found in the cell soma and on the cell surface evenly distributed within the pc/dc-complex. (**C**) GlyRs are evenly distributed in the pc/dc-complex and the staining pattern shows the typical receptor spots. A–C: Scale bar 50 µm. (**D**) Merged display of anti-gephyrin and anti-GlyR staining on the cellular level. Anti-gephyrin staining is found throughout the whole cytoplasm, while anti-GlyR staining is limited to concentrated spots on the cell surface (double arrow heads). Inset is magnified in G. (**E**) Staining pattern of gephyrin on the cellular level displayed in green. Inset is magnified in H. (**F**) Staining pattern of anti-GlyR on the cellular level displayed in red. Inset is magnified in I. D–F: Scale bar 10 µm. (**G**) Magnifying inset (displayed in **D**) of the concentrated Glycin receptors (red) in colocalization with gephyrin labelled cells (green). (**H**) Magnifying inset displayed in (**E**) of gephyrin labelled cells (green). (**I**) Magnifying inset (displayed in **F**) of concentrated Glycin receptors (red). G–H: Scale bar 5 µm. Images were created with ImageJ 1.52n (Fiji, available from https://fiji.sc/)^38^.

Cells labelled with anti-gephyrin and anti-GlyR antibodies were found throughout the whole pc/dc-complex tissue (Fig. S3, Fig. 4A–C). We found gephyrin in the cytoplasm and on the surface of the cell soma (Fig. 4D–F). GlyRs are limited to the cell surface in colocalization with gephyrin (Fig. 4G–I).

#### Reorganization of inhibitory synaptic sites

**Figure 5:**
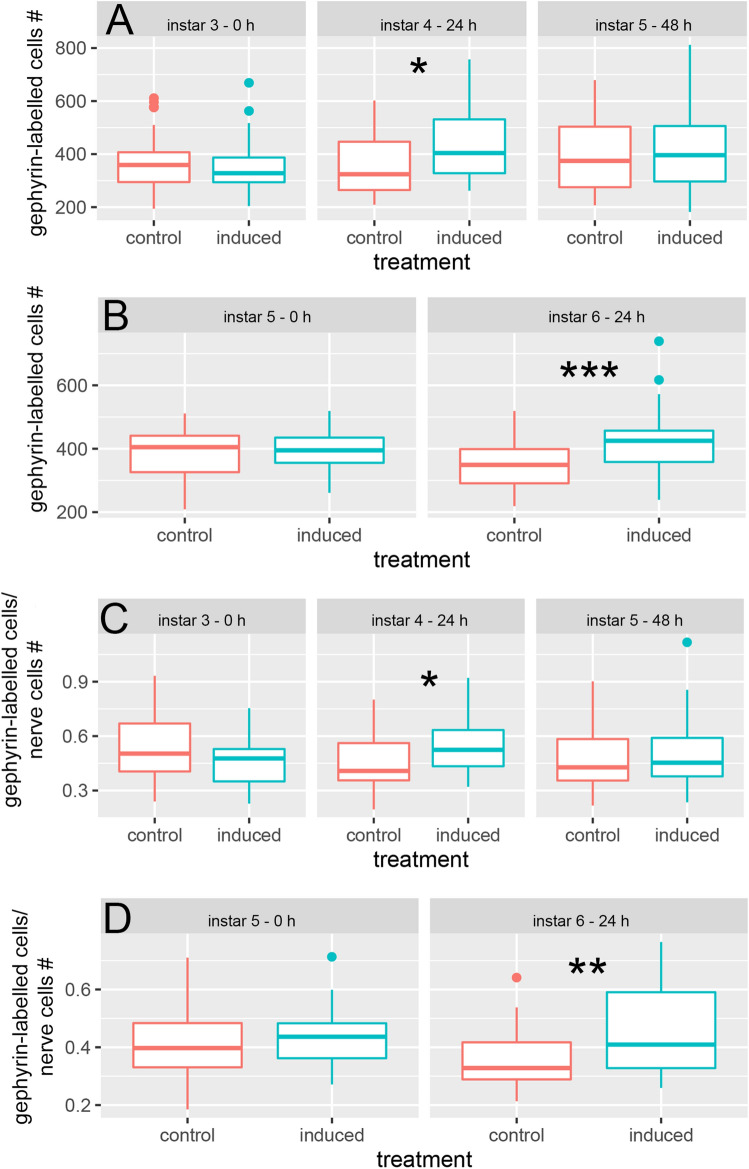
Functional plasticity underlying morphological defense expression. (**A**) When exposed to predators from the 3rd instar onwards, the number of gephyrin-labelled cells is significantly increased after 24 h predator-exposure compared to the control (*P* = 0.013; t =  − 2.56; df = 57) and to the 3rd instar (ANOVA: F_(2;97)_ = 3.12 *P* = 0.049 Tukey: *P* = 0.039). (**B**) When exposed to predators from the 5^th^ instar onwards, the number of gephyrin-labelled cells is significantly increased after 24 h predator exposure compared to the control (*P* ≤ 0.001; t =  − 3.63; df = 44). (**C**) The number of gephyrin-labelled cells per nerve cells is significantly increased compared to the control after 24 h predator exposure, when the animals were exposed to predators from the 3rd instar onwards (*P* = 0.011; t =  − 2.65; df = 57). (**D**) When exposed to predators from the 5th instar, the ratio of gephyrin-labelled cells/nerve cells is significantly increased compared to the control after 24 h predator exposure (*P* ≤ 0.001; t =  − 3.41; df = 44). (**A**, **C)** Alpha corrected levels of significance: *P ≤ 0.017. **(B**, **D)** Alpha corrected levels of significance: **P ≤ 0.005; ***P ≤ 0.0005. Boxplots show median (middle line), 25th to 75th percentiles (box) and whiskers show highest and lowest value within 1.5× the interquartile range (1.5× below the 25th percentile and 1.5× above the 75th percentile), respectively. Dots indicate outliers > 1.5× the interquartile range.

In control animals the number of gephyrin-labelled cells did not change over time (Fig. 5A; Tables S1, S2). In individuals that we exposed to predators from the 3rd instar onwards, the number of gephyrin-labelled cells increased significantly from a mean of 352.62 ± 105.41 cells (0 h) to 440.62 ± 138.76 cells within 24 h (Fig. 5A; Tables S1, S2). In 48 h predator exposed specimens the number of gephyrin-labelled cells remained constant in comparison to 24 h exposed specimens (Fig. 5A; Tables S1, S2). At 0 h no significant difference in the number of gephyrin-labelled cells was detected between the control and predator-exposed group (Fig. 5A; Table S3). After 24 h predator exposure individuals showed a significantly increased number of gephyrin-labelled cells compared to the control (Fig. 5A; Table S1). After 48 h predator exposure no significant difference in the number of gephyrin-labelled cells was detected between the two treatments (Fig. 5A; Table S1). In individuals that we exposed from the 5th instar onwards the number of gephyrin-labelled cells in predator-exposed *D. longicephala* was significantly increased in comparison to the control after 24 h predator exposure (Fig. 5B, Table S4).

At the beginning of the experiment and prior to predator exposure (0 h), we detected no significant differences between the gephyrin-labelled cells per absolute cell number in the two treatments (Fig. 5C; Table S3). After 24 h predator-exposed individuals showed a significantly higher ratio than controls (Fig. 5C; Table S3). 48 h after initial predator exposure no significant differences between the treatments were detected (Fig. 5C; Table S3).

The ratio of gephyrin-labelled cells/neurons in individuals that we exposed from the 5th instar onwards, was significantly increased in predator-exposed individuals compared to controls after 24 h predator exposure as well (Fig. 5D; Table S5).

## Discussion

### Inducible defense expression

When we expose *D. longicephala* to predators in the 3rd instar, we observe a tendency towards larger crests in the 4th instar and significant crest expression in the 5th instar. We observe a similar result when we expose the animals in the 5th instar, upon which crests are also significantly expressed 24 h later. This indicates that predator exposure gradually induced crest expression within a short time frame of 24 h, so that defenses become effective in a fast manner as reported previously^15^.

### Structural plasticity in the pc/dc-complex

#### Volumetric changes of the pc/dc-complex and the OG

As the main cognitive functions are performed by the protocerebrum, we focused on the pc/dc-complex. The mean volume of naïve *D. longicephala* pc/dc-complexes increases 1.4-fold over the observed instars showing the highest volume in the 6th instar with ~ 350,000 µm^3^. Larvae of the fruit fly *Drosophila* show a total brain volume of ~ 800,000 µm^3^ ^44^ and adult flies even ~ 80 Mio µm^3^ ^45^, with a body size similar to that of adult *D. longicephala*. When exposed to predators for 24 h and 48 h, *D. longicephala* showed significantly larger pc/dc-complex volumes than the respective controls. We detected this predator-induced volumetric increase independent of the *Daphnias’* age. Therefore, the growth of the morphological defense is accompanied with the growth of the pc/dc-complex. Our results show that the volume of predator-induced animals’ pc/dc-complexes more than doubles (2.2-fold) within the first 48 h of predator exposure from ~ 250,000 µm^3^ to a volume of ~ 565,000 µm^3^. This means that predator-exposed individuals show a 60% larger pc/dc-complex compared to controls after 48 h.

In *Drosophila,* different brain structures have been observed to increase in response to diversified environments that are e.g. enriched with food vials, odor sources and conspecifics. The *Drosophila* central brain is probably best comparable to the pc/dc- complex in *Daphnia,* and this structure increased its volume by 7.5% when encountering complex environments for 19 days^46^. The largest volumetric increase of 21% was observed in the calyx, a part of the mushroom bodies^26^. The volumetric increase of the *Daphnia* pc/dc-complex is three times as high as the largest volumetric increase in *Drosophila* brains. Furthermore, the increase in the *Daphnia* pc/dc-complex appears much faster and reflects a strong structural plasticity in a very short time. This may be an important factor when coping with predators, as the defenses need to be expressed in due time so that strong and rapid structural plasticity is probably selected for. Mechanistically, such a high degree of volumetric expansion can only be achieved if either the absolute number of cells, the cell size or intercellular distances increase. Larger intercellular distances provide additional space for cell- cell connections in the form of dendrites that expand the cell surface. It is reasonable to suggest that the basis of such novel cell–cell connections is established during the first 24 h of predator perception. Since we detected no volumetric changes in the OG between the observed treatments, the functional plasticity observed in the pc/dc-complex has to be a result of predator-exposure. This supports the hypothesis that the pc/dc-complex plays a central role in inducible defense expression.

#### Nerve cell numbers in the pc/dc-complex

The 1.4-fold pc/dc-complex volume increase that we observe with age and the 2.2 fold increase that we observe with predator exposure must originate from some kind of gain in neuronal tissue. Therefore, we determined the overall number of cells, irrespective of their type, in the pc/dc-complex. With increasing instars, we detected a 1.2-fold increase in cell numbers within the pc/dc-complex. Cell numbers increased from ~ 750 cells in the 3rd instar to ~ 900 cells in the 6th instar. So, with age the animals obtain more cells in this central complex. As the animals grow continuously throughout life, it is possible that the number of cells in the brain continues to increase. In fact, a life-long addition of new neurons, e.g. in the olfactory pathways of insects^47^ and crustaceans^48–50^, has already been reported. Similarly, the continuous increase in body size requires the addition of new receptor neurons to cope with the increased surface area of the body, the length of the appendages or the size of the compound eye^51–53^. This probably also leads to the observed volumetric increase in the OG with increasing age (i.e. ~ 2.9-fold increase in control and predator exposed *D. longicephala*). The 1.23-fold increase in the number of brain cells in the pc/dc-complex lies within the range of the volumetric changes across instars (1.4-fold), so that there may be a direct correlation between volume and cell number. However, this correlation only holds true when comparing successive instars. Between control and predator-exposed animals, we did not observe that the 2.2-fold increase in brain volume is due to an increase in the cell number (i.e. only 1.23-fold). Therefore, the predator-induced increase in pc/dc-complex volume must rely on other factors, e.g. less dense brain tissues and/or larger neurons in predator-exposed *D.* *longicephala*. In fact, in rats it has already been reported that an enriched and demanding environment leads to increased brain volume based on reduced cell density and increased cell size but the number of neurons in the brain tissue was not affected (reviewed in^32^). It can be speculated that larger intercellular clefts provide more space for structures like dendrites and synapses, while bigger cell bodies provide a larger surface area on which more synapses can attach. This has been observed in the visual cortex of rats^19^. Individuals exposed to a complex environment had larger neurons, allowing the attachment of 20% more synapses per neuron than rats from less complex environments. It was hypothesized that a larger neuron size may improve cellular stability and efficiency. Likewise, larger cells may also be required to ensure the metabolic support of more extensive dendritic fields^19^. As synaptic density remains unaltered in plastic brain tissue^32^, an increased neuron size is necessary for the attachment of additional synapses. Therefore, the here observed increase in pc/dc-complex volume is not correlated with an increase of cell numbers but must depend on an increase in inter-neuronal connections. We hypothesize that there is a predator-induced rewiring of inter-neuronal connections i.e. through the development of new dendrites, increasing intercellular distances and thus the overall volume. Likewise, we anticipate that neuron size is increased, which enables the attachment of more synapses and enhances cell–cell stability. This may contribute to the formation of a dedicated neuronal circuitry that is associated with predator perception.

### Functional plasticity in the pc/dc-complex

#### Reorganization of inhibitory synaptic sites

In order to investigate potential remodeling and/or emergence of a dedicated cell circuitry, we focused on the number and distribution of inhibitory cell synapses indicated by the scaffolding protein gephyrin. The staining pattern of gephyrin shows intensive markings in the cytoplasm and on the surface of the cell soma, which is typical for this antibody and explained by the fact that gephyrin is involved in post-Golgi transport and cell surface delivery of GlyRs^36^. In contrast, GlyRs are observed with a typical dot-like staining pattern on the cell membranes. Gephyrin was found in colocalization with GlyRs, and all cells that stained with the anti-GlyR antibody showed a colocalization with the gephyrin antibody. This colocalization clearly demonstrates that also in *Daphnia*, gephyrin is the anchoring protein for receptors like GlyRs at inhibitory PSDs. The fact that we observe gephyrin-labelled cells that do not co-localize with GlyRs indicates that gephyrin also anchors other inhibitory receptors like the GABA_A_ receptor known from other species^33^. Unfortunately, we so far did not find a specific GABA_A_R antibody to test for this co-localization and therefore this awaits further investigation.

Both, gephyrin as well as GlyRs are homogeneously distributed throughout the pc/dc-complex. We did not observe any kind of neuropil concentric localization of gephyrin that would point to a specialized brain area. This pattern maintained also throughout predator exposure. However, after 24 h of predator exposure, the number of gephyrin-labelled cells in the pc/dc-complex is significantly increased compared to controls. As the number of neurons is stable between treatments, the ratio of gephyrin-labelled cells per neuron is also significantly higher in predator-exposed individuals after 24 h. This coincides with the onset of crest development and is again age independent. An increased number of cells expressing gephyrin indicates the presence of more inhibitory synaptic connections, as gephyrin anchors and clusters GABA_A_Rs and GlyRs at the postsynaptic site^35^.

Our findings show that the number of cells possessing inhibitory synapses in pc/dc-complexes of predator-exposed *D.* *longicephala* increases in line with the morphological defense expression. It is possible that glycinergic signals play a role in the pathway underlying predator perception. Controlling the excitability of nerve cells is central to ensuring normal brain function^54^, and increased inhibition could thus lead to a more directional excitability that focuses on crucial functions. Hence, adapted inhibition patterns in the *Daphnia* brain might be a central element of predator perception. While the involvement of glycinergic responses is still unknown, the involvement of GABAergic signaling in predator perception has been demonstrated in *D. pulex* responding to fish and *Chaoborus* predation^13^*.* It is very likely that the emergence of additional GABA_A_Rs occurs upon the perception of predator cues. Functionally, GABA_A_Rs are a central component of synaptic plasticity by ensuring that glutamatergic N-methyl-D-aspartate receptors (NMDARs) are predominantly activated during high frequency synaptic transmission only^55^. Especially, NMDARs play a fundamental role in long-term potentiation (LTP) and long-term depression (LTD), two common forms of synaptic plasticity responsible for fostering cell–cell connections^56,57^. It is therefore possible that such mechanisms play a role in deciphering and determining the predation risk amongst the diversity of environmental cues.

We only observe this increase in gephyrin-labelled cells in the instar that coincides with the onset of defense expression i.e. 24 h of predator exposure. In the later stage, we do not see differences in the number of gephyrin-labelled cells in comparison to the control group. However, although defense expression persists in the following instar i.e. after 48 h predator exposure, we detect no further changes in gephyrin abundance between control and predator-exposed individuals, which is an effect of a slight and not significant increase of the total number of nerve cells in the controls. With the increase in the total number of cells, also the proportion of gephyrin-labelled cells increases. Even if this increase is not significant, it diminishes the differences between the predator-induced and control groups in 5th instar specimens and is a result of the gradual growth of the animals. Nevertheless, the number of gephyrin-labelled cells remains at a constant level, so that there are no differences between 4th and 5th instar predator exposed specimens. We hypothesize that there is a dedicated rewiring event during the initial phase of predator exposure. Once this rewiring is accomplished, this new neuronal circuitry is manifested, indicated by the unchanged number of gephyrin-labelled cells of 48 h exposed specimens. It remains to be determined whether this pattern will persist at later stages and will require a longer observation period in future experiments.

Taken together, the increase in gephyrin expression supports our hypothesis that putatively larger cells enable the enrichment of synaptic sites. Thus, the increased gephyrin abundance might not only indicate a higher number of cells possessing inhibitory synapses but also more inhibitory synapses per neuron within pc/dc-complexes of predator-induced individuals. By this, the directional excitability might be further enhanced as the activation of inhibitory receptors has also been found to interfere with glutamatergic inputs if the respective synapses are located on the same dendritic branch^58,59^. The increase or reduction of activity at synapses is achieved by adjustments of the postsynaptic response, like modulation of receptor availability at the post synaptic site^60,61^. Highly mobile GABA_A_R forms allow trafficking of inhibitory receptors, which has been postulated to be important for the modulation and expression of various forms of synaptic plasticity^56,62^. Additionally, GABA_A_R mediated transmission itself can be modified as a result of plasticity in inhibitory synapses^63^. In this regard, evidence for the potential modification of GABA_A_Rs has been found in form of increasing receptor numbers^56,64^.

Although we focused on the rewiring of the inhibitory nervous system, inhibition and excitation act in concert to form directed and dedicated neuronal signal patterns. It is thus very likely to suggest that there is also a rewiring of excitatory synapses accompanying the signal integration of predator perception.

In conclusion, our findings suggest three mechanisms that are involved in signal integration of predator-specific chemical cues in the *Daphnia* pc/dc-complex: 1.) An increase in the pc/dc-complex volume provides space for more cell–cell connections 2.) The formation of more inhibitory synaptic connections that control cellular activity, increasing directionality of neuronal signaling 3.) The rewiring of a neuronal circuitry that is associated with predator perception. Taken together, our study provides new insights into how organisms interpret environmental challenges and transform these into phenotypic adaptations.

## Supplementary Information

Supplementary Information.

## Data Availability

Data associated with this manuscript is given in the supplements.
