# Supplementary material for: Brain volume increase and neuronal plasticity underly predator-induced morphological defense expression in Daphnia longicephala
Source: Sci Rep. 2021 Jun 15;11:12612. doi: 10.1038/s41598-021-92052-y (PMC8206331; doi:10.1038/s41598-021-92052-y)
Supplement: Supplementary file 1 — Supplementary Information. [file 41598_2021_92052_MOESM1_ESM.docx]

**Brain volume increase and neuronal plasticity underly predator-induced morphological defense expression in *Daphnia longicephala***

Graeve A^1†^, Ioannidou I^1†^, Reinhard J^2^, Görl D. M.^1^, Faissner A^2^ and Weiss LC^1*^

**Supplementary material**

As both antibodies have not been tested in *Daphnia*, we first aimed to validate antibody specificity. Only the anti-glycine receptor antibody is suitable for Western blot (WB) applications, but not the anti-gephyrin antibody. Therefore, we validated the specificity of the anti-glycine receptor antibody and performed co-immunostaining of anti-glycine and anti-gephyrin. The staining pattern showed a clear co-localization of both antibodies, which indicates that gephyrin is used as an anchoring protein of glycine receptors in *Daphnia*.

**Western Blot**

We dissected 150 *Daphnia* brains and homogenized the tissue in lysis buffer (60 mM n-octyl-β-D-glucopyranoside, 50 mM sodium acetate, 50 mM Tris chloride (pH 8.0) and 2 M urea) containing a protease inhibitor cocktail (Sigma Aldrich). Afterwards, we centrifuged the protein homogenate at 14.000 x g at 4°C for 30 min. The supernatant was used to determine protein concentration with a BCA Protein Assay kit (Pierce; Thermo Fisher Scientific, Rockford, IL, USA) following the manufacturer’s instructions. Next, 4x SDS sample buffer was added to each protein sample (40 μg). We then denaturized the samples at 95°C for 5 min. For separation by SDS-PAGE 4-10% polyacrylamide gradient gels were used. Via Western blotting, proteins were transferred to polyvinylidene difluoride (PVDF) membranes (Roth, Karlsruhe, Germany). Membranes were blocked at room temperature for 1 h in 5% w/v milk powder in Tris-buffered saline (TBS) and Tween 20 (TBST). Primary anti-glycine receptor (Cat. No. 146 011; Synaptic Systems, Germany; RRID:AB_887722) (1:500) and anti-actin Ab-5 (Cat. No. 612 656; Biosciences; RRID:AB_2289199) (1:1000) antibodies both raised in mouse were diluted in blocking solution and applied on the PVDF membranes overnight. Following washing in TBST, horseradish peroxidase-(HRP-) coupled secondary antibodies were diluted in blocking solution (for GlyR detection 1:3000, for Actin detection 1:5000) and applied to the membrane. After incubation at room temperature for 1 h, membranes were washed. Next, an ECL Substrate (Bio-Rad Laboratories GmbH, München, Germany) was mixed 1:1 and added to the membranes for 30 min to detect protein bands. Protein bands were documented using the MicroChemi Chemilumiscence Reader (Biostep, Burkhardtsdorf, Germany).


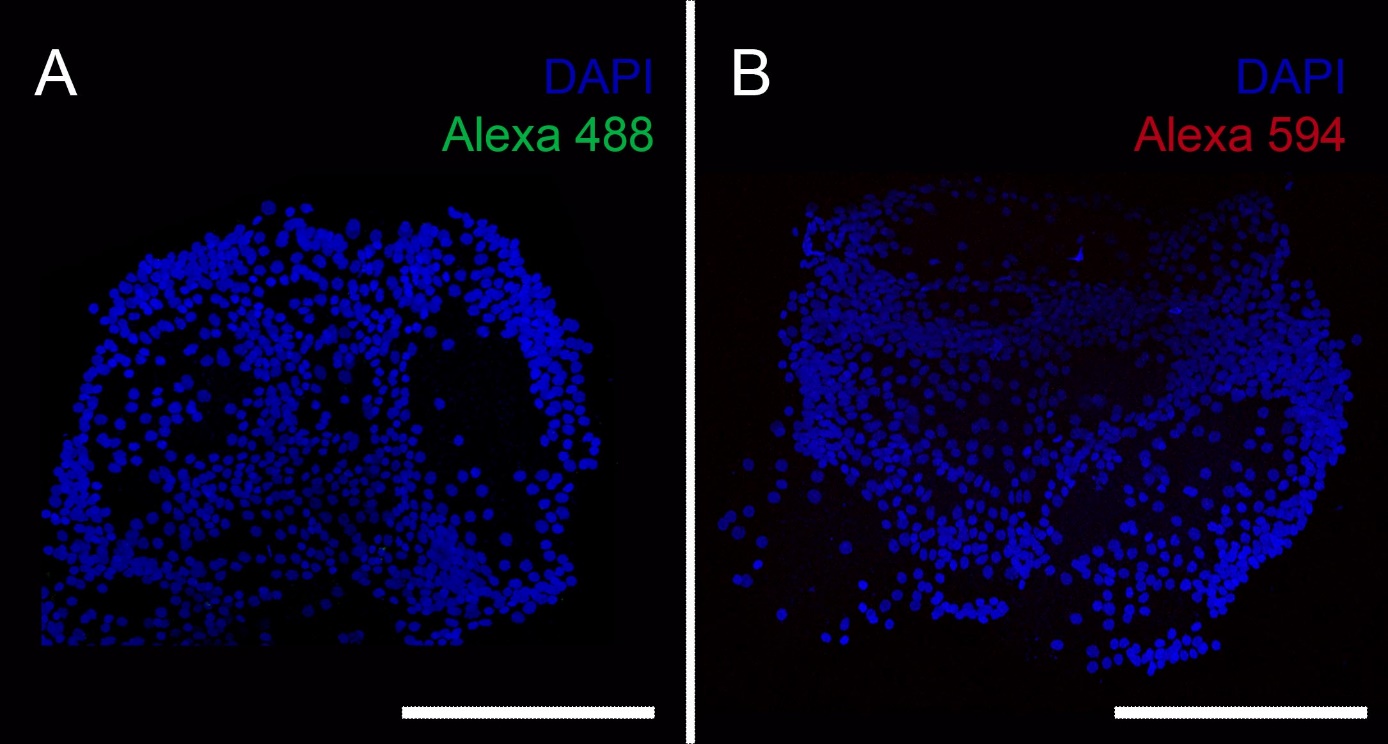


**Figure S1:** Negative control of secondary antibodies on *D. longicephala* pc/ dc-complex. A: Merged display of secondary antibody anti-mouse Alexa 488 and DAPI staining pattern. There is no unspecific binding of the anti-mouse Alexa 488 to the *D. longicephala* pc/ dc-complex tissue. B: Merged display of secondary antibody anti-rabbit Alexa 594 and DAPI staining pattern. No unspecific binding of the anti-rabbit Alexa 594 to the D. longicephala pc/ dc-complex tissue is detected. Brains are displayed in the frontal orientation; top rostral, bottom: caudal.


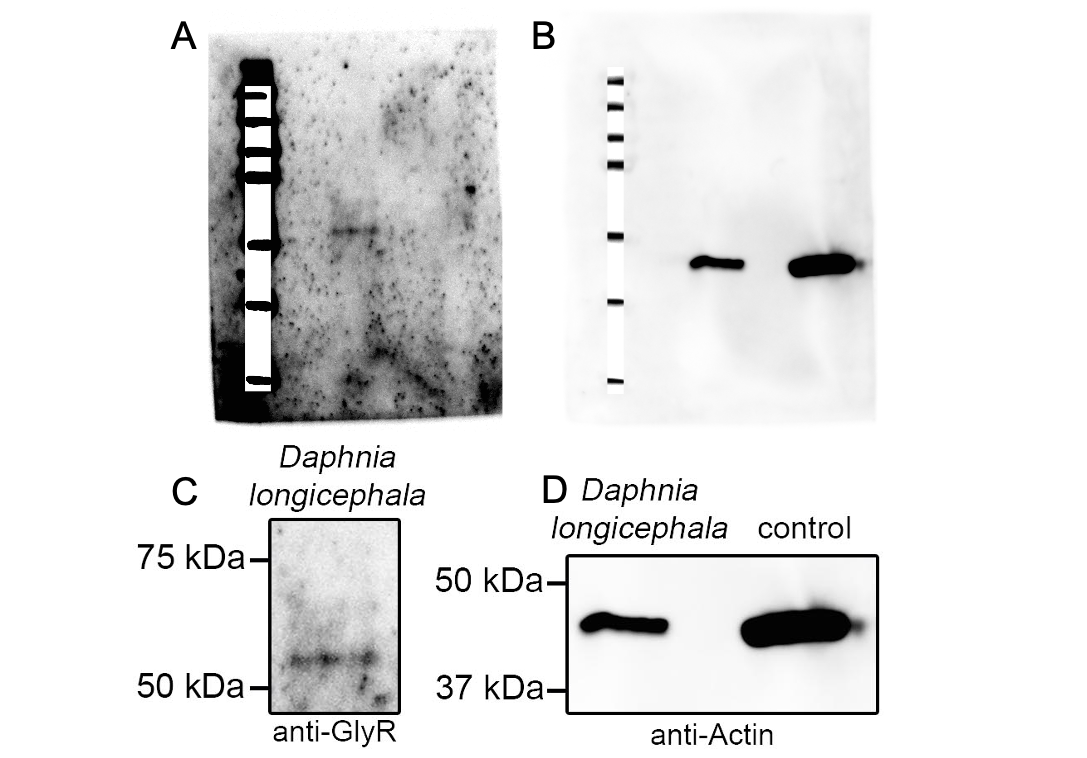


**Figure S2**: Western Blot of anti-glycine receptor and anti-actin on *D. longicephala* brain tissue. A: Original blot image for glycine receptor detection. B: Original blot image for Actin detection. C: We detect a band at approximately 55 kDa for the glycine receptor. D: The actin control creates a band at approximately 42 kDa in the *D. longicephala* brain tissue (left) and in the mouse neurospheres (right) that served as a positive control.

**
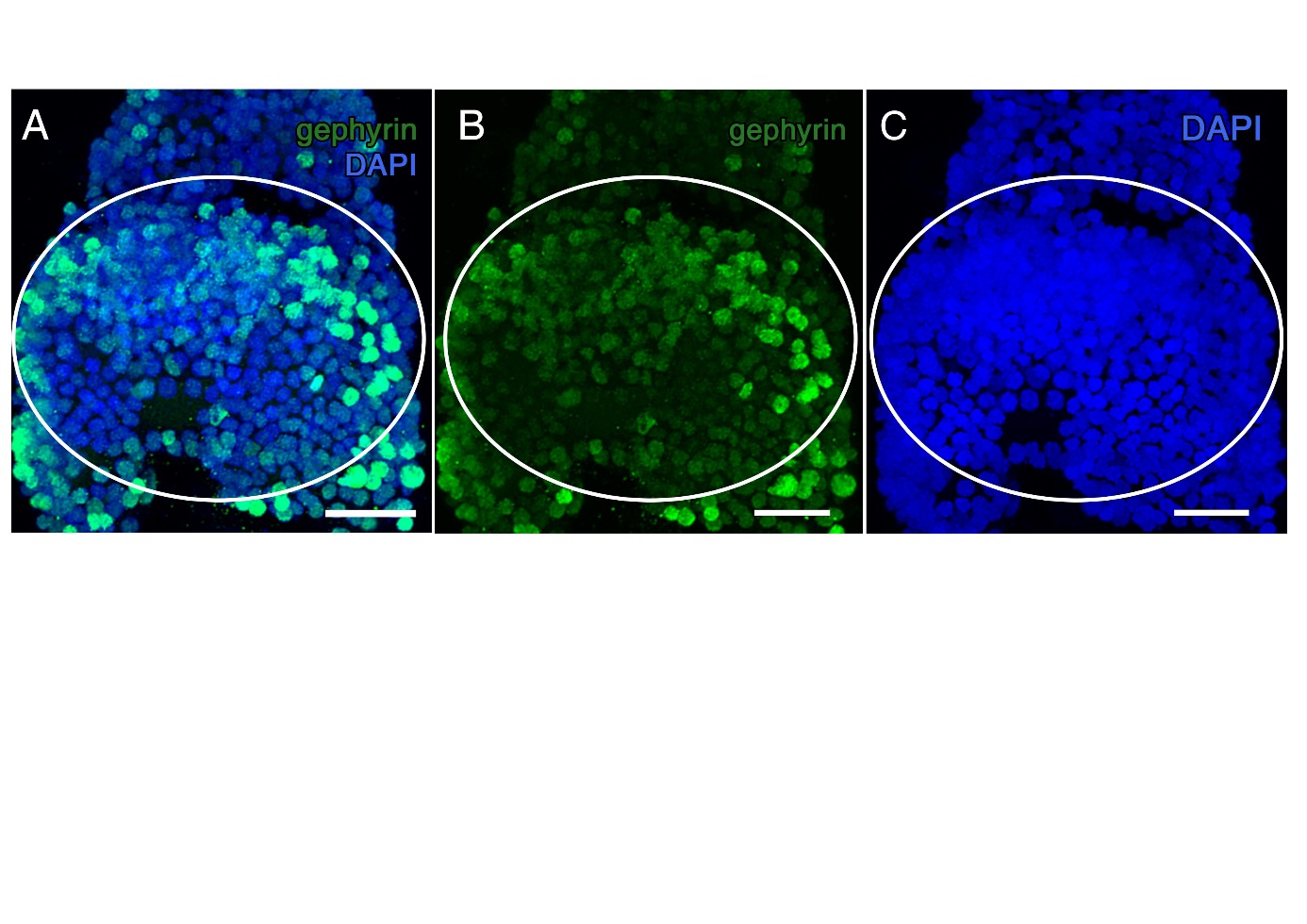
**

**Figure S3:** Co-localization of anti-gephyrin and DAPI. (A) Merged image of anti-gephyrin and DAPI. Anti-gephyrin staining is found throughout the whole brain tissue, but not in every cell. We detect a co-localization of DAPI and anti-gephyrin, with gephyrin mostly located on the nuclear surface and in the cytoplasm. (B) Anti-gephyrin staining pattern displayed in green. (C) Nuclear DAPI staining is displayed in blue. White circle marks the pc/dc-complex; scale bar 50 µm. Brains are displayed in the frontal orientation; top rostral, bottom: caudal.

Table S1: One-way ANOVA on crest height, pc/ dc-complex volume, OG volume, log(nerve cells), log(gephyrin-labelled cells), log(gephyrin-labelled cells/nerve cells) in instars 3 to 5 in control or predator-exposed *D. longicephala*, initially exposed in the 3^rd^ instar. Abbreviations: SS, square sum; MS, mean square

| Crest height | | | | | | | | | |
| --- | --- | --- | --- | --- | --- | --- | --- | --- | --- |
| Effect | Treatment | SS | Degree Of Freedom | MS | F | *P* | Partial eta-squared | Non-centrality | Observed power (alpha=0.05) |
| Intercept | control | 3,774,209 | 1 | 3,774,209 | 3,089.95 | 0.0001 | 0.969 | 3,089.95 | 1.00 |
| Instar |  | 284 | 2 | 142 | 0.12 | 0.890 | 0.002 | 0.23 | 0.07 |
| Error |  | 119,702 | 98 | 1,221 |  |  |  |  |  |
| Intercept | induced | 4,535,613 | 1 | 4,535,613 | 1,412.39 | 0.0001 | 0.936 | 1,412.39 | 1.00 |
| Instar |  | 30,673 | 2 | 15,336 | 4.78 | 0.011 | 0.090 | 9.55 | 0.78 |
| Error |  | 311,496 | 97 | 3,211 |  |  |  |  |  |
| pc/dc volume | | | | | | | | | |
| Intercept | control | 3.57E+20 | 1 | 3.57E+20 | 157.23 | 0.0001 | 0.762 | 157.23 | 1.00 |
| Instar |  | 2.56E+18 | 2 | 1.28E+18 | 0.56 | 0.573 | 0.022 | 1.13 | 0.14 |
| Error |  | 1.11E+20 | 49 | 2.27E+18 |  |  |  |  |  |
| Intercept | induced | 5.33E+20 | 1 | 5.33E+20 | 195.75 | 0.0001 | 0.852 | 195.75 | 1.00 |
| Instar |  | 2.91E+19 | 2 | 1.46E+19 | 5.35 | 0.010 | 0.239 | 10.71 | 0.80 |
| Error |  | 9.25E+19 | 34 | 2.72E+18 |  |  |  |  |  |
| OG volume | | | | | | | | | |
| Intercept | control | 3.52E+11 | 1 | 3.52E+11 | 109.01 | 0.0001 | 0.893 | 109.01 | 1.00 |
| Instar |  | 6.22E+10 | 2 | 3.11E+10 | 9.63 | 0.003 | 0.597 | 19.25 | 0.94 |
| Error |  | 4.20E+10 | 13 | 3.23E+09 |  |  |  |  |  |
| Intercept | induced | 4.95E+11 | 1 | 4.95E+11 | 166.28 | 0.0001 | 0.922 | 166.28 | 1.00 |
| Instar |  | 7.19E+10 | 2 | 3.60E+10 | 12.07 | 0.001 | 0.633 | 24.14 | 0.98 |
| Error |  | 4.17E+10 | 14 | 2.98E+09 |  |  |  |  |  |
| log(nerve cells) | | | | | | | | | |
| Intercept | control | 816.19 | 1 | 816.19 | 43,801.81 | 0.0001 | 1.00 | 43,801.81 | 1.00 |
| Instar |  | 0.10 | 2 | 0.05 | 2.64 | 0.077 | 0.05 | 5.27 | 0.51 |
| Error |  | 1.83 | 98 | 0.02 |  |  |  |  |  |
| Intercept | induced | 814.90 | 1 | 814.90 | 56,132.75 | 0.0001 | 1.00 | 56,132.75 | 1.00 |
| Instar |  | 0.01 | 2 | 0.00 | 0.20 | 0.821 | 0.00 | 0.39 | 0.08 |
| Error |  | 1.41 | 97 | 0.01 |  |  |  |  |  |
| log(gephyrin-labelled cells) | | | | | | | | | |
| Intercept | control | 634.78 | 1 | 634.78 | 35,767.39 | 0.0001 | 1.00 | 35,767.39 | 1.00 |
| Instar |  | 0.02 | 2 | 0.01 | 0.44 | 0.644 | 0.01 | 0.89 | 0.12 |
| Error |  | 1.74 | 98 | 0.02 |  |  |  |  |  |
| Intercept | induced | 645.58 | 1 | 645.58 | 31,033.35 | 0.0001 | 1.00 | 31,033.35 | 1.00 |
| Instar |  | 0.13 | 2 | 0.06 | 3.12 | 0.049 | 0.06 | 6.25 | 0.59 |
| Error |  | 2.02 | 97 | 0.02 |  |  |  |  |  |
| log(Gephyrin/ nerve cells) | | | | | | | | | |
| Intercept | control | 11.38 | 1 | 11.38 | 477.12 | 0.0001 | 0.83 | 477.12 | 1 |
| Instar |  | 0.10 | 2 | 0.05 | 2.14 | 0.123 | 0.04 | 4.29 | 0.43 |
| Error |  | 2.34 | 98 | 0.02 |  |  |  |  |  |
| Intercept | induced | 9.85 | 1 | 9.85 | 509.66 | 0.0001 | 0.84 | 509.66 | 1 |
| Instar |  | 0.12 | 2 | 0.06 | 3.01 | 0.054 | 0.06 | 6.03 | 0.57 |
| Error |  | 1.87 | 97 | 0.02 |  |  |  |  |  |

Table S2: Post-hoc Tukey-test on crest height, pc/ dc-complex volume, log-nerve cells, log-gephyrin-labelled cells, gephyrin-labelled cells/nerve cells in instars 3 to 5 in control or predator-exposed *D. longicephala*, initially exposed in the 3^rd^ instar.

| Crest height | | | | |
| --- | --- | --- | --- | --- |
| treatment | instar | 3 | 4 | 5 |
| control | 3 |  | 0.880 | 0.981 |
|  | 4 | 0.880 |  | 0.962 |
|  | 5 | 0.981 | 0.962 |  |
| induced | 3 |  | 0.173 | 0.007 |
|  | 4 | 0.173 |  | 0.503 |
|  | 5 | 0.007 | 0.503 |  |
| pc/dc volume | | | | |
| treatment | instar | 3 | 4 | 5 |
| control | 3 |  | 0.786 | 0.545 |
|  | 4 | 0.786 |  | 0.941 |
|  | 5 | 0.545 | 0.941 |  |
| induced | 3 |  | 0.016 | 0.031 |
|  | 4 | 0.016 |  | 1.000 |
|  | 5 | 0.031 | 1.000 |  |
| OG volume | | | | |
| treatment | instar | 3 | 4 | 5 |
| control | 3 |  | 0.173 | 0.002 |
|  | 4 | 0.173 |  | 0.082 |
|  | 5 | 0.002 | 0.082 |  |
| induced | 3 |  | 0.063 | 0.001 |
|  | 4 | 0.063 |  | 0.035 |
|  | 5 | 0.001 | 0.035 |  |
| log(nerve cells) | | | | |
| treatment | instar | 3 | 4 | 5 |
| control | 3 |  | 0.305 | 0.076 |
|  | 4 | 0.305 |  | 0.767 |
|  | 5 | 0.076 | 0.767 |  |
| induced | 3 |  | 0.987 | 0.823 |
|  | 4 | 0.987 |  | 0.905 |
|  | 5 | 0.823 | 0.905 |  |
| log(gephyrin-labelled cells) | | | | |
| treatment | instar | 3 | 4 | 5 |
| control | 3 |  | 0.809 | 0.917 |
|  | 4 | 0.809 |  | 0.623 |
|  | 5 | 0.917 | 0.623 |  |
| induced | 3 |  | 0.039 | 0.261 |
|  | 4 | 0.039 |  | 0.499 |
|  | 5 | 0.261 | 0.499 |  |
| log(gephyrin/nerve cells) | | | | |
| treatment | instar | 3 | 4 | 5 |
| control | 3 |  | 0.161 | 0.249 |
|  | 4 | 0.161 |  | 0.981 |
|  | 5 | 0.249 | 0.981 |  |
| induced | 3 |  | 0.043 | 0.506 |
|  | 4 | 0.043 |  | 0.278 |
|  | 5 | 0.506 | 0.278 |  |

Table S3: T-test on crest height, pc/ dc-complex volume, OG volume, log(nerve cells), log(gephyrin-labelled cells), log(gephyrin-labelled cells/ nerve cells) in control and predator-exposed *D. longicephala*, exposed from the 3^rd^ instar to the 5^th^ instar. Abbreviations: SS, square sum; MS, mean square

| Variable | instar | Mean control | Mean induced | t-value | df | *P* | Valid N control | Valid  N induced | SD control | SD induced | F-ratio |
| --- | --- | --- | --- | --- | --- | --- | --- | --- | --- | --- | --- |
| Crest height | 3 | 194.88 | 193.19 | 0.21 | 70 | 0.834 | 43 | 29 | 31.45 | 36.52 | 1.35 |
| Volume [µm^3^] |  | 249,348.7 | 247,623.7 | -0.05 | 27 | 0.957 | 15 | 14 | 70,955.12 | 97,067.05 | 1.87 |
| OG volume [µm^3^] |  | 75,837.76 | 89,783.36 | -1.06 | 8 | 0.321 | 5 | 5 | 21,080.22 | 20,623.36 | 1.04 |
| log(#DAPI-labelled cells) |  | 2.86 | 2.85 | -1.55 | 70 | 0.126 | 43 | 29 | 0.11 | 0.09 | 1.60 |
| log(#gephyrin-labelled cells) |  | 2.55 | 2.53 | 0.83 | 70 | 0.411 | 43 | 29 | 0.12 | 0.12 | 1.11 |
| log(#gephyrin/# DAPI -labelled cells) |  | -0.30 | -0.36 | 1.77 | 70 | 0.082 | 43 | 29 | 0.15 | 0.14 | 1.17 |
| Crest height | 4 | 198.89 | 220.08 | -1.91 | 57 | 0.061 | 30 | 29 | 37.68 | 46.97 | 1.55 |
| Volume [µm^3^] |  | 310,676.8 | 451,761.4 | 2.73 | 28 | 0.011 | 15 | 15 | 136,421.9 | 146,165.9 | 1.15 |
| OG volume [µm^3^] |  | 144,814.0 | 169,535.8 | -0.73 | 10 | 0.480 | 5 | 7 | 70,597.18 | 46,988.7 | 2.23 |
| log(#DAPI-labelled cells) |  | 2.90 | 2.90 | 0.18 | 57 | 0.859 | 30 | 29 | 0.13 | 0.07 | 3.29 |
| log(#gephyrin-labelled cells) |  | 2.53 | 2.62 | -2.56 | 57 | 0.013 | 30 | 29 | 0.14 | 0.13 | 1.06 |
| log(#gephyrin/# DAPI -labelled cells) |  | -0.37 | -0.27 | -2.65 | 57 | 0.011 | 30 | 29 | 0.15 | 0.13 | 1.37 |
| Crest height | 5 | 196.46 | 235.42 | -2.64 | 68 | 0.010 | 28 | 42 | 37.02 | 71.97 | 3.78 |
| Volume [µm^3^] |  | 353,098.4 | 565,640.5 | 2.59 | 33 | 0.014 | 22 | 13 | 142,285.7 | 34,0501.1 | 5.73 |
| OG volume [µm^3^] |  | 226,191.2 | 259,253.9 | -0.76 | 9 | 0.470 | 6 | 5 | 63,729.89 | 81,789.8 | 1.65 |
| log(#DAPI-labelled cells) |  | 2.93 | 2.91 | 0.44 | 68 | 0.659 | 28 | 42 | 0.17 | 0.16 | 1.14 |
| log(#gephyrin-labelled cells) |  | 2.57 | 2.58 | -0.47 | 68 | 0.640 | 28 | 42 | 0.15 | 0.16 | 1.16 |
| log(#gephyrin/# DAPI -labelled cells) |  | -0.36 | -0.32 | -0.95 | 68 | 0.347 | 28 | 42 | 0.17 | 0.15 | 1.30 |

Table S4: T-test on crest height, pc/ dc-complex volume, log(nerve cells), log(gephyrin-labelled cells), log(gephyrin-labelled cells/nerve cells) in control and predator-exposed *D. longicephala* in instars 5 and 6, initially exposed in the 5^th^ instar.

| Variable | instar | Mean 5^th^ instar | Mean 6^th^ instar | t-value | df | *P* | Valid N 5^th^ instar | Valid  N 6^th^ instar | SD  N 5^th^ instar | SD  N 6^th^ instar | F-ratio |
| --- | --- | --- | --- | --- | --- | --- | --- | --- | --- | --- | --- |
| Crest height | control | 171.27 | 174.97 | -0.39 | 51 | 0.700 | 32 | 21 | 37.05 | 28.93 | 1.64 |
| Volume µm^3^ |  | 338,462.98 | 310,848.17 | 0.37 | 7 | 0.721 | 5 | 4 | 101,146.23 | 122,657.01 | 1.47 |
| log(#DAPI-labelled cells) |  | 2.99 | 2.98 | 0.29 | 51 | 0.773 | 32 | 21 | 0.09 | 0.10 | 1.16 |
| log(#gephyrin-labelled cells) |  | 2.58 | 2.59 | -0.43 | 51 | 0.668 | 32 | 21 | 0.09 | 0.07 | 1.42 |
| log(#gephyrin/# DAPI labelled cells) |  | -0.41 | -0.39 | -0.58 | 51 | 0.563 | 32 | 21 | 0.12 | 0.09 | 1.68 |
| Crest height | induced | 169.90 | 206.55 | -4.18 | 44 | 0.000 | 25 | 21 | 31.90 | 26.63 | 1.44 |
| Volume µm^3^ |  | 350,290.39 | 424,134.15 | -2.19 | 12 | 0.049 | 6 | 8 | 81,781.86 | 43,543.65 | 3.53 |
| log(#DAPIi labelled cells) |  | 2.99 | 2.97 | 0.89 | 44 | 0.376 | 25 | 21 | 0.10 | 0.09 | 1.29 |
| log(#gephyrin labelled cells) |  | 2.53 | 2.64 | -3.63 | 44 | 0.001 | 25 | 21 | 0.10 | 0.09 | 1.05 |
| log(#gephyrin/#DAPI labelled cells) |  | -0.46 | -0.33 | -3.50 | 44 | 0.001 | 25 | 21 | 0.12 | 0.13 | 1.27 |

Table S5: T-test on crest height, log-nerve cells, log-gephyrin-labelled cells, gephyrin-labelled cells/ nerve cells in instars 5 to 6 in control or predator-exposed *D. longicephala*, initially exposed in the 5^th^ instar.

| Variable | Treatment | Mean control | Mean induced | t-value | df | *P* | Valid N control | Valid  N induced | SD control | SD induced | F-ratio |
| --- | --- | --- | --- | --- | --- | --- | --- | --- | --- | --- | --- |
| Crest height | 5 | 171.27 | 169.90 | 0.15 | 55 | 0.884 | 32 | 25 | 37.05 | 31.90 | 1.35 |
| Volume µm^3^ |  | 338,462.98 | 350,290.39 | -0.21 | 9 | 0.835 | 5 | 6 | 101,146.23 | 81,781.86 | 1.53 |
| log(#DAPI-labelled cells) |  | 2.99 | 2.99 | -0.19 | 55 | 0.848 | 32 | 25 | 0.09 | 0.10 | 1.31 |
| log(#gephyrin-labelled cells) |  | 2.58 | 2.53 | 1.78 | 55 | 0.081 | 32 | 25 | 0.09 | 0.10 | 1.18 |
| log(#gephyrin-/#DAPI- labelled cells) |  | -0.41 | -0.46 | 1.55 | 55 | 0.126 | 32 | 25 | 0.12 | 0.12 | 1.01 |
| Crest height | 6 | 174.97 | 206.55 | -3.68 | 40 | 0.001 | 21 | 21 | 28.93 | 26.63 | 1.18 |
| Volume µm^3^ |  | 310,848.17 | 424,134.15 | -2.42 | 10 | 0.036 | 4 | 8 | 122,657.01 | 43,543.65 | 7.93 |
| log(#DAPI-labelled cells) |  | 2.98 | 2.97 | 0.46 | 40 | 0.646 | 21 | 21 | 0.10 | 0.09 | 1.14 |
| log(#gephyrin-labelled cells) |  | 2.59 | 2.64 | -1.86 | 40 | 0.070 | 21 | 21 | 0.07 | 0.09 | 1.60 |
| log(#gephyrin-/#DAPI-labelled cells) |  | -0.39 | -0.33 | -1.78 | 40 | 0.082 | 21 | 21 | 0.09 | 0.13 | 2.11 |

Table S6: Data associated with figure 2. Log values are rounded to 2 decimals.

| instar | treatment | Crest height  (µm) | log (crest height) | DAPI | log(dapi) | Gephyrin | log(Gephyrin) | Gephyrin/DAPI | log (Gephyrin/DAPI) |
| --- | --- | --- | --- | --- | --- | --- | --- | --- | --- |
| 3 | control | 173.40 | 2.24 | 1131.00 | 3.05 | 271.00 | 2.43 | 0.24 | -0.62 |
| 3 | control | 189.30 | 2.28 | 1116.00 | 3.05 | 314.00 | 2.50 | 0.28 | -0.55 |
| 3 | control | 137.30 | 2.14 | 911.00 | 2.96 | 267.00 | 2.43 | 0.29 | -0.53 |
| 3 | control | 175.60 | 2.24 | 654.00 | 2.82 | 194.00 | 2.29 | 0.30 | -0.53 |
| 3 | control | 175.40 | 2.24 | 844.00 | 2.93 | 258.00 | 2.41 | 0.31 | -0.51 |
| 3 | control | 215.30 | 2.33 | 929.00 | 2.97 | 299.00 | 2.48 | 0.32 | -0.49 |
| 3 | control | 170.70 | 2.23 | 1102.00 | 3.04 | 375.00 | 2.57 | 0.34 | -0.47 |
| 3 | control | 198.00 | 2.30 | 809.00 | 2.91 | 285.00 | 2.45 | 0.35 | -0.45 |
| 3 | control | 217.00 | 2.34 | 632.00 | 2.80 | 243.00 | 2.39 | 0.38 | -0.42 |
| 3 | control | 267.60 | 2.43 | 741.00 | 2.87 | 290.00 | 2.46 | 0.39 | -0.41 |
| 3 | control | 206.90 | 2.32 | 704.00 | 2.85 | 285.00 | 2.45 | 0.40 | -0.39 |
| 3 | control | 170.30 | 2.23 | 772.00 | 2.89 | 313.00 | 2.50 | 0.41 | -0.39 |
| 3 | control | 210.80 | 2.32 | 1002.00 | 3.00 | 409.00 | 2.61 | 0.41 | -0.39 |
| 3 | control | 199.30 | 2.30 | 671.00 | 2.83 | 286.00 | 2.46 | 0.43 | -0.37 |
| 3 | control | 167.30 | 2.22 | 809.00 | 2.91 | 348.00 | 2.54 | 0.43 | -0.37 |
| 3 | control | 181.90 | 2.26 | 889.00 | 2.95 | 390.00 | 2.59 | 0.44 | -0.36 |
| 3 | control | 188.50 | 2.28 | 812.00 | 2.91 | 359.00 | 2.56 | 0.44 | -0.35 |
| 3 | control | 216.10 | 2.33 | 801.00 | 2.90 | 367.00 | 2.56 | 0.46 | -0.34 |
| 3 | control | 187.00 | 2.27 | 771.00 | 2.89 | 376.00 | 2.58 | 0.49 | -0.31 |
| 3 | control | 220.70 | 2.34 | 602.00 | 2.78 | 301.00 | 2.48 | 0.50 | -0.30 |
| 3 | control | 182.50 | 2.26 | 512.00 | 2.71 | 257.00 | 2.41 | 0.50 | -0.30 |
| 3 | control | 185.70 | 2.27 | 715.00 | 2.85 | 360.00 | 2.56 | 0.50 | -0.30 |
| 3 | control | 207.20 | 2.32 | 755.00 | 2.88 | 382.00 | 2.58 | 0.51 | -0.30 |
| 3 | control | 192.20 | 2.28 | 746.00 | 2.87 | 378.00 | 2.58 | 0.51 | -0.30 |
| 3 | control | 161.80 | 2.21 | 764.00 | 2.88 | 395.00 | 2.60 | 0.52 | -0.29 |
| 3 | control | 215.40 | 2.33 | 653.00 | 2.81 | 339.00 | 2.53 | 0.52 | -0.28 |
| 3 | control | 272.40 | 2.44 | 470.00 | 2.67 | 257.00 | 2.41 | 0.55 | -0.26 |
| 3 | control | 247.20 | 2.39 | 912.00 | 2.96 | 507.00 | 2.71 | 0.56 | -0.25 |
| 3 | control | 177.40 | 2.25 | 549.00 | 2.74 | 308.00 | 2.49 | 0.56 | -0.25 |
| 3 | control | 139.10 | 2.14 | 514.00 | 2.71 | 309.00 | 2.49 | 0.60 | -0.22 |
| 3 | control | 173.40 | 2.24 | 653.00 | 2.81 | 413.00 | 2.62 | 0.63 | -0.20 |
| 3 | control | 160.20 | 2.20 | 904.00 | 2.96 | 577.00 | 2.76 | 0.64 | -0.19 |
| 3 | control | 178.20 | 2.25 | 578.00 | 2.76 | 405.00 | 2.61 | 0.70 | -0.15 |
| 3 | control | 201.40 | 2.30 | 848.00 | 2.93 | 611.00 | 2.79 | 0.72 | -0.14 |
| 3 | control | 175.20 | 2.24 | 828.00 | 2.92 | 597.00 | 2.78 | 0.72 | -0.14 |
| 3 | control | 195.60 | 2.29 | 647.00 | 2.81 | 469.00 | 2.67 | 0.72 | -0.14 |
| 3 | control | 248.90 | 2.40 | 699.00 | 2.84 | 510.00 | 2.71 | 0.73 | -0.14 |
| 3 | control | 140.90 | 2.15 | 473.00 | 2.67 | 358.00 | 2.55 | 0.76 | -0.12 |
| 3 | control | 188.40 | 2.28 | 736.00 | 2.87 | 579.00 | 2.76 | 0.79 | -0.10 |
| 3 | control | 190.30 | 2.28 | 463.00 | 2.67 | 372.00 | 2.57 | 0.80 | -0.10 |
| 3 | control | 255.30 | 2.41 | 498.00 | 2.70 | 434.00 | 2.64 | 0.87 | -0.06 |
| 3 | control | 226.80 | 2.36 | 359.00 | 2.56 | 333.00 | 2.52 | 0.93 | -0.03 |
| 3 | control | 196.10 | 2.29 | 621.00 | 2.79 | 579.00 | 2.76 | 0.93 | -0.03 |
| 4 | control | 248.30 | 2.39 | 1260.00 | 3.10 | 247.00 | 2.39 | 0.20 | -0.71 |
| 4 | control | 203.70 | 2.31 | 1063.00 | 3.03 | 209.00 | 2.32 | 0.20 | -0.71 |
| 4 | control | 208.60 | 2.32 | 986.00 | 2.99 | 301.00 | 2.48 | 0.31 | -0.52 |
| 4 | control | 186.90 | 2.27 | 780.00 | 2.89 | 269.00 | 2.43 | 0.34 | -0.46 |
| 4 | control | 175.30 | 2.24 | 895.00 | 2.95 | 309.00 | 2.49 | 0.35 | -0.46 |
| 4 | control | 143.50 | 2.16 | 1266.00 | 3.10 | 440.00 | 2.64 | 0.35 | -0.46 |
| 4 | control | 133.50 | 2.13 | 647.00 | 2.81 | 225.00 | 2.35 | 0.35 | -0.46 |
| 4 | control | 181.50 | 2.26 | 767.00 | 2.88 | 271.00 | 2.43 | 0.35 | -0.45 |
| 4 | control | 283.90 | 2.45 | 706.00 | 2.85 | 257.00 | 2.41 | 0.36 | -0.44 |
| 4 | control | 199.70 | 2.30 | 646.00 | 2.81 | 239.00 | 2.38 | 0.37 | -0.43 |
| 4 | control | 251.30 | 2.40 | 599.00 | 2.78 | 223.00 | 2.35 | 0.37 | -0.43 |
| 4 | control | 201.10 | 2.30 | 1012.00 | 3.01 | 377.00 | 2.58 | 0.37 | -0.43 |
| 4 | control | 252.60 | 2.40 | 781.00 | 2.89 | 298.00 | 2.47 | 0.38 | -0.42 |
| 4 | control | 197.00 | 2.29 | 626.00 | 2.80 | 250.00 | 2.40 | 0.40 | -0.40 |
| 4 | control | 126.40 | 2.10 | 913.00 | 2.96 | 371.00 | 2.57 | 0.41 | -0.39 |
| 4 | control | 189.30 | 2.28 | 1189.00 | 3.08 | 487.00 | 2.69 | 0.41 | -0.39 |
| 4 | control | 172.80 | 2.24 | 1245.00 | 3.10 | 510.00 | 2.71 | 0.41 | -0.39 |
| 4 | control | 213.50 | 2.33 | 985.00 | 2.99 | 427.00 | 2.63 | 0.43 | -0.36 |
| 4 | control | 154.20 | 2.19 | 1107.00 | 3.04 | 500.00 | 2.70 | 0.45 | -0.35 |
| 4 | control | 193.80 | 2.29 | 600.00 | 2.78 | 280.00 | 2.45 | 0.47 | -0.33 |
| 4 | control | 207.40 | 2.32 | 567.00 | 2.75 | 273.00 | 2.44 | 0.48 | -0.32 |
| 4 | control | 149.70 | 2.18 | 828.00 | 2.92 | 461.00 | 2.66 | 0.56 | -0.25 |
| 4 | control | 179.80 | 2.25 | 1069.00 | 3.03 | 602.00 | 2.78 | 0.56 | -0.25 |
| 4 | control | 238.90 | 2.38 | 777.00 | 2.89 | 438.00 | 2.64 | 0.56 | -0.25 |
| 4 | control | 216.40 | 2.34 | 756.00 | 2.88 | 449.00 | 2.65 | 0.59 | -0.23 |
| 4 | control | 215.40 | 2.33 | 873.00 | 2.94 | 523.00 | 2.72 | 0.60 | -0.22 |
| 4 | control | 174.30 | 2.24 | 404.00 | 2.61 | 263.00 | 2.42 | 0.65 | -0.19 |
| 4 | control | 246.00 | 2.39 | 482.00 | 2.68 | 362.00 | 2.56 | 0.75 | -0.12 |
| 4 | control | 233.10 | 2.37 | 431.00 | 2.63 | 339.00 | 2.53 | 0.79 | -0.10 |
| 4 | control | 188.80 | 2.28 | 714.00 | 2.85 | 572.00 | 2.76 | 0.80 | -0.10 |
| 5 | control | 237.40 | 2.38 | 1469.00 | 3.17 | 319.00 | 2.50 | 0.22 | -0.66 |
| 5 | control | 260.10 | 2.42 | 1047.00 | 3.02 | 230.00 | 2.36 | 0.22 | -0.66 |
| 5 | control | 240.20 | 2.38 | 1130.00 | 3.05 | 267.00 | 2.43 | 0.24 | -0.63 |
| 5 | control | 203.20 | 2.31 | 1390.00 | 3.14 | 340.00 | 2.53 | 0.24 | -0.61 |
| 5 | control | 193.80 | 2.29 | 1320.00 | 3.12 | 423.00 | 2.63 | 0.32 | -0.49 |
| 5 | control | 222.00 | 2.35 | 660.00 | 2.82 | 217.00 | 2.34 | 0.33 | -0.48 |
| 5 | control | 174.40 | 2.24 | 879.00 | 2.94 | 306.00 | 2.49 | 0.35 | -0.46 |
| 5 | control | 208.10 | 2.32 | 596.00 | 2.78 | 213.00 | 2.33 | 0.36 | -0.45 |
| 5 | control | 194.50 | 2.29 | 1034.00 | 3.01 | 395.00 | 2.60 | 0.38 | -0.42 |
| 5 | control | 189.00 | 2.28 | 965.00 | 2.98 | 369.00 | 2.57 | 0.38 | -0.42 |
| 5 | control | 185.10 | 2.27 | 1281.00 | 3.11 | 496.00 | 2.70 | 0.39 | -0.41 |
| 5 | control | 168.50 | 2.23 | 1276.00 | 3.11 | 530.00 | 2.72 | 0.42 | -0.38 |
| 5 | control | 211.70 | 2.33 | 1211.00 | 3.08 | 514.00 | 2.71 | 0.42 | -0.37 |
| 5 | control | 144.80 | 2.16 | 1173.00 | 3.07 | 500.00 | 2.70 | 0.43 | -0.37 |
| 5 | control | 223.70 | 2.35 | 962.00 | 2.98 | 413.00 | 2.62 | 0.43 | -0.37 |
| 5 | control | 233.30 | 2.37 | 849.00 | 2.93 | 368.00 | 2.57 | 0.43 | -0.36 |
| 5 | control | 208.10 | 2.32 | 448.00 | 2.65 | 207.00 | 2.32 | 0.46 | -0.34 |
| 5 | control | 233.70 | 2.37 | 1141.00 | 3.06 | 529.00 | 2.72 | 0.46 | -0.33 |
| 5 | control | 220.10 | 2.34 | 504.00 | 2.70 | 247.00 | 2.39 | 0.49 | -0.31 |
| 5 | control | 187.00 | 2.27 | 879.00 | 2.94 | 462.00 | 2.66 | 0.53 | -0.28 |
| 5 | control | 246.30 | 2.39 | 429.00 | 2.63 | 249.00 | 2.40 | 0.58 | -0.24 |
| 5 | control | 206.80 | 2.32 | 519.00 | 2.72 | 308.00 | 2.49 | 0.59 | -0.23 |
| 5 | control | 126.30 | 2.10 | 1001.00 | 3.00 | 608.00 | 2.78 | 0.61 | -0.22 |
| 5 | control | 167.00 | 2.22 | 853.00 | 2.93 | 545.00 | 2.74 | 0.64 | -0.19 |
| 5 | control | 186.80 | 2.27 | 403.00 | 2.61 | 278.00 | 2.44 | 0.69 | -0.16 |
| 5 | control | 186.10 | 2.27 | 508.00 | 2.71 | 380.00 | 2.58 | 0.75 | -0.13 |
| 5 | control | 116.80 | 2.07 | 762.00 | 2.88 | 679.00 | 2.83 | 0.89 | -0.05 |
| 5 | control | 126.00 | 2.10 | 600.00 | 2.78 | 541.00 | 2.73 | 0.90 | -0.04 |
| 3 | induced | 192.80 | 2.29 | 904.00 | 2.96 | 206.00 | 2.31 | 0.23 | -0.64 |
| 3 | induced | 190.00 | 2.28 | 846.00 | 2.93 | 206.00 | 2.31 | 0.24 | -0.61 |
| 3 | induced | 188.80 | 2.28 | 1003.00 | 3.00 | 258.00 | 2.41 | 0.26 | -0.59 |
| 3 | induced | 173.80 | 2.24 | 752.00 | 2.88 | 204.00 | 2.31 | 0.27 | -0.57 |
| 3 | induced | 193.40 | 2.29 | 1057.00 | 3.02 | 294.00 | 2.47 | 0.28 | -0.56 |
| 3 | induced | 210.20 | 2.32 | 957.00 | 2.98 | 328.00 | 2.52 | 0.34 | -0.47 |
| 3 | induced | 198.00 | 2.30 | 731.00 | 2.86 | 255.00 | 2.41 | 0.35 | -0.46 |
| 3 | induced | 175.30 | 2.24 | 862.00 | 2.94 | 302.00 | 2.48 | 0.35 | -0.46 |
| 3 | induced | 226.40 | 2.35 | 1089.00 | 3.04 | 414.00 | 2.62 | 0.38 | -0.42 |
| 3 | induced | 162.20 | 2.21 | 858.00 | 2.93 | 339.00 | 2.53 | 0.40 | -0.40 |
| 3 | induced | 214.30 | 2.33 | 712.00 | 2.85 | 291.00 | 2.46 | 0.41 | -0.39 |
| 3 | induced | 169.10 | 2.23 | 676.00 | 2.83 | 295.00 | 2.47 | 0.44 | -0.36 |
| 3 | induced | 154.20 | 2.19 | 855.00 | 2.93 | 377.00 | 2.58 | 0.44 | -0.36 |
| 3 | induced | 186.50 | 2.27 | 669.00 | 2.83 | 304.00 | 2.48 | 0.45 | -0.34 |
| 3 | induced | 150.50 | 2.18 | 671.00 | 2.83 | 320.00 | 2.51 | 0.48 | -0.32 |
| 3 | induced | 244.50 | 2.39 | 655.00 | 2.82 | 313.00 | 2.50 | 0.48 | -0.32 |
| 3 | induced | 163.50 | 2.21 | 798.00 | 2.90 | 386.00 | 2.59 | 0.48 | -0.32 |
| 3 | induced | 189.60 | 2.28 | 746.00 | 2.87 | 363.00 | 2.56 | 0.49 | -0.31 |
| 3 | induced | 165.50 | 2.22 | 642.00 | 2.81 | 318.00 | 2.50 | 0.50 | -0.31 |
| 3 | induced | 218.30 | 2.34 | 552.00 | 2.74 | 275.00 | 2.44 | 0.50 | -0.30 |
| 3 | induced | 268.00 | 2.43 | 1019.00 | 3.01 | 517.00 | 2.71 | 0.51 | -0.29 |
| 3 | induced | 143.90 | 2.16 | 853.00 | 2.93 | 451.00 | 2.65 | 0.53 | -0.28 |
| 3 | induced | 243.00 | 2.39 | 801.00 | 2.90 | 433.00 | 2.64 | 0.54 | -0.27 |
| 3 | induced | 183.80 | 2.26 | 792.00 | 2.90 | 429.00 | 2.63 | 0.54 | -0.27 |
| 3 | induced | 178.30 | 2.25 | 927.00 | 2.97 | 564.00 | 2.75 | 0.61 | -0.22 |
| 3 | induced | 170.20 | 2.23 | 594.00 | 2.77 | 367.00 | 2.56 | 0.62 | -0.21 |
| 3 | induced | 281.20 | 2.45 | 605.00 | 2.78 | 387.00 | 2.59 | 0.64 | -0.19 |
| 3 | induced | 130.50 | 2.12 | 478.00 | 2.68 | 360.00 | 2.56 | 0.75 | -0.12 |
| 3 | induced | 236.60 | 2.37 | 889.00 | 2.95 | 670.00 | 2.83 | 0.75 | -0.12 |
| 4 | induced | 212.80 | 2.33 | 943.00 | 2.97 | 302.00 | 2.48 | 0.32 | -0.49 |
| 4 | induced | 292.70 | 2.47 | 806.00 | 2.91 | 278.00 | 2.44 | 0.34 | -0.46 |
| 4 | induced | 148.80 | 2.17 | 950.00 | 2.98 | 328.00 | 2.52 | 0.35 | -0.46 |
| 4 | induced | 234.00 | 2.37 | 716.00 | 2.85 | 262.00 | 2.42 | 0.37 | -0.44 |
| 4 | induced | 293.30 | 2.47 | 1077.00 | 3.03 | 401.00 | 2.60 | 0.37 | -0.43 |
| 4 | induced | 179.80 | 2.25 | 916.00 | 2.96 | 346.00 | 2.54 | 0.38 | -0.42 |
| 4 | induced | 300.80 | 2.48 | 786.00 | 2.90 | 340.00 | 2.53 | 0.43 | -0.36 |
| 4 | induced | 213.00 | 2.33 | 726.00 | 2.86 | 315.00 | 2.50 | 0.43 | -0.36 |
| 4 | induced | 193.80 | 2.29 | 823.00 | 2.92 | 381.00 | 2.58 | 0.46 | -0.33 |
| 4 | induced | 234.00 | 2.37 | 889.00 | 2.95 | 423.00 | 2.63 | 0.48 | -0.32 |
| 4 | induced | 289.60 | 2.46 | 604.00 | 2.78 | 295.00 | 2.47 | 0.49 | -0.31 |
| 4 | induced | 174.20 | 2.24 | 789.00 | 2.90 | 404.00 | 2.61 | 0.51 | -0.29 |
| 4 | induced | 253.80 | 2.40 | 532.00 | 2.73 | 275.00 | 2.44 | 0.52 | -0.29 |
| 4 | induced | 213.30 | 2.33 | 809.00 | 2.91 | 424.00 | 2.63 | 0.52 | -0.28 |
| 4 | induced | 297.30 | 2.47 | 557.00 | 2.75 | 292.00 | 2.47 | 0.52 | -0.28 |
| 4 | induced | 155.50 | 2.19 | 994.00 | 3.00 | 531.00 | 2.73 | 0.53 | -0.27 |
| 4 | induced | 182.50 | 2.26 | 726.00 | 2.86 | 402.00 | 2.60 | 0.55 | -0.26 |
| 4 | induced | 257.90 | 2.41 | 901.00 | 2.95 | 517.00 | 2.71 | 0.57 | -0.24 |
| 4 | induced | 234.60 | 2.37 | 842.00 | 2.93 | 507.00 | 2.71 | 0.60 | -0.22 |
| 4 | induced | 271.70 | 2.43 | 690.00 | 2.84 | 418.00 | 2.62 | 0.61 | -0.22 |
| 4 | induced | 189.20 | 2.28 | 732.00 | 2.86 | 459.00 | 2.66 | 0.63 | -0.20 |
| 4 | induced | 155.60 | 2.19 | 873.00 | 2.94 | 553.00 | 2.74 | 0.63 | -0.20 |
| 4 | induced | 170.00 | 2.23 | 572.00 | 2.76 | 396.00 | 2.60 | 0.69 | -0.16 |
| 4 | induced | 218.10 | 2.34 | 826.00 | 2.92 | 579.00 | 2.76 | 0.70 | -0.15 |
| 4 | induced | 239.30 | 2.38 | 889.00 | 2.95 | 678.00 | 2.83 | 0.76 | -0.12 |
| 4 | induced | 178.70 | 2.25 | 842.00 | 2.93 | 643.00 | 2.81 | 0.76 | -0.12 |
| 4 | induced | 168.60 | 2.23 | 719.00 | 2.86 | 573.00 | 2.76 | 0.80 | -0.10 |
| 4 | induced | 201.20 | 2.30 | 842.00 | 2.93 | 757.00 | 2.88 | 0.90 | -0.05 |
| 4 | induced | 228.20 | 2.36 | 759.00 | 2.88 | 699.00 | 2.84 | 0.92 | -0.04 |
| 5 | induced | 191.80 | 2.28 | 1135.00 | 3.05 | 267.00 | 2.43 | 0.24 | -0.63 |
| 5 | induced | 250.30 | 2.40 | 1070.00 | 3.03 | 302.00 | 2.48 | 0.28 | -0.55 |
| 5 | induced | 214.20 | 2.33 | 869.00 | 2.94 | 259.00 | 2.41 | 0.30 | -0.53 |
| 5 | induced | 174.30 | 2.24 | 1672.00 | 3.22 | 503.00 | 2.70 | 0.30 | -0.52 |
| 5 | induced | 181.40 | 2.26 | 1235.00 | 3.09 | 390.00 | 2.59 | 0.32 | -0.50 |
| 5 | induced | 177.10 | 2.25 | 1215.00 | 3.08 | 390.00 | 2.59 | 0.32 | -0.49 |
| 5 | induced | 186.20 | 2.27 | 1267.00 | 3.10 | 440.00 | 2.64 | 0.35 | -0.46 |
| 5 | induced | 301.00 | 2.48 | 521.00 | 2.72 | 182.00 | 2.26 | 0.35 | -0.46 |
| 5 | induced | 280.90 | 2.45 | 729.00 | 2.86 | 266.00 | 2.42 | 0.36 | -0.44 |
| 5 | induced | 252.50 | 2.40 | 979.00 | 2.99 | 361.00 | 2.56 | 0.37 | -0.43 |
| 5 | induced | 231.60 | 2.36 | 592.00 | 2.77 | 223.00 | 2.35 | 0.38 | -0.42 |
| 5 | induced | 242.30 | 2.38 | 664.00 | 2.82 | 254.00 | 2.40 | 0.38 | -0.42 |
| 5 | induced | 145.70 | 2.16 | 1159.00 | 3.06 | 457.00 | 2.66 | 0.39 | -0.40 |
| 5 | induced | 335.20 | 2.53 | 809.00 | 2.91 | 323.00 | 2.51 | 0.40 | -0.40 |
| 5 | induced | 161.80 | 2.21 | 892.00 | 2.95 | 358.00 | 2.55 | 0.40 | -0.40 |
| 5 | induced | 200.30 | 2.30 | 945.00 | 2.98 | 383.00 | 2.58 | 0.41 | -0.39 |
| 5 | induced | 161.90 | 2.21 | 1213.00 | 3.08 | 501.00 | 2.70 | 0.41 | -0.38 |
| 5 | induced | 159.20 | 2.20 | 952.00 | 2.98 | 405.00 | 2.61 | 0.43 | -0.37 |
| 5 | induced | 315.10 | 2.50 | 639.00 | 2.81 | 282.00 | 2.45 | 0.44 | -0.36 |
| 5 | induced | 228.70 | 2.36 | 659.00 | 2.82 | 295.00 | 2.47 | 0.45 | -0.35 |
| 5 | induced | 271.10 | 2.43 | 1124.00 | 3.05 | 509.00 | 2.71 | 0.45 | -0.34 |
| 5 | induced | 180.90 | 2.26 | 886.00 | 2.95 | 402.00 | 2.60 | 0.45 | -0.34 |
| 5 | induced | 146.40 | 2.17 | 1101.00 | 3.04 | 514.00 | 2.71 | 0.47 | -0.33 |
| 5 | induced | 195.70 | 2.29 | 755.00 | 2.88 | 369.00 | 2.57 | 0.49 | -0.31 |
| 5 | induced | 171.20 | 2.23 | 621.00 | 2.79 | 305.00 | 2.48 | 0.49 | -0.31 |
| 5 | induced | 342.90 | 2.54 | 418.00 | 2.62 | 209.00 | 2.32 | 0.50 | -0.30 |
| 5 | induced | 213.80 | 2.33 | 1018.00 | 3.01 | 550.00 | 2.74 | 0.54 | -0.27 |
| 5 | induced | 141.30 | 2.15 | 1024.00 | 3.01 | 555.00 | 2.74 | 0.54 | -0.27 |
| 5 | induced | 307.40 | 2.49 | 346.00 | 2.54 | 190.00 | 2.28 | 0.55 | -0.26 |
| 5 | induced | 233.50 | 2.37 | 359.00 | 2.56 | 204.00 | 2.31 | 0.57 | -0.25 |
| 5 | induced | 429.50 | 2.63 | 895.00 | 2.95 | 519.00 | 2.72 | 0.58 | -0.24 |
| 5 | induced | 230.30 | 2.36 | 1343.00 | 3.13 | 797.00 | 2.90 | 0.59 | -0.23 |
| 5 | induced | 175.20 | 2.24 | 1021.00 | 3.01 | 621.00 | 2.79 | 0.61 | -0.22 |
| 5 | induced | 158.80 | 2.20 | 916.00 | 2.96 | 568.00 | 2.75 | 0.62 | -0.21 |
| 5 | induced | 290.20 | 2.46 | 507.00 | 2.71 | 324.00 | 2.51 | 0.64 | -0.19 |
| 5 | induced | 381.30 | 2.58 | 710.00 | 2.85 | 507.00 | 2.71 | 0.71 | -0.15 |
| 5 | induced | 301.00 | 2.48 | 565.00 | 2.75 | 404.00 | 2.61 | 0.72 | -0.15 |
| 5 | induced | 228.90 | 2.36 | 598.00 | 2.78 | 453.00 | 2.66 | 0.76 | -0.12 |
| 5 | induced | 226.50 | 2.36 | 946.00 | 2.98 | 724.00 | 2.86 | 0.77 | -0.12 |
| 5 | induced | 382.80 | 2.58 | 578.00 | 2.76 | 489.00 | 2.69 | 0.85 | -0.07 |
| 5 | induced | 301.30 | 2.48 | 490.00 | 2.69 | 419.00 | 2.62 | 0.86 | -0.07 |
| 5 | induced | 186.10 | 2.27 | 727.00 | 2.86 | 812.00 | 2.91 | 1.12 | 0.05 |

Table S7: Data associated with figure 3. Log values are rounded to 2 decimals.

| instar | treatment | Crest height  (µm) | log (crest height) | DAPI | log(dapi) | Gephyrin | log(Gephyrin) | Gephyrin/DAPI | log (Gephyrin/DAPI) |
| --- | --- | --- | --- | --- | --- | --- | --- | --- | --- |
| 5 | control | 179.80 | 2.25 | 896.00 | 2.95 | 511.00 | 2.71 | 0.57 | -0.24 |
| 5 | control | 163.50 | 2.21 | 1095.00 | 3.04 | 406.00 | 2.61 | 0.37 | -0.43 |
| 5 | control | 122.20 | 2.09 | 851.00 | 2.93 | 304.00 | 2.48 | 0.36 | -0.45 |
| 5 | control | 150.90 | 2.18 | 891.00 | 2.95 | 472.00 | 2.67 | 0.53 | -0.28 |
| 5 | control | 147.50 | 2.17 | 1064.00 | 3.03 | 326.00 | 2.51 | 0.31 | -0.51 |
| 5 | control | 191.10 | 2.28 | 1296.00 | 3.11 | 408.00 | 2.61 | 0.31 | -0.50 |
| 5 | control | 178.20 | 2.25 | 1018.00 | 3.01 | 406.00 | 2.61 | 0.40 | -0.40 |
| 5 | control | 158.20 | 2.20 | 961.00 | 2.98 | 418.00 | 2.62 | 0.43 | -0.36 |
| 5 | control | 170.30 | 2.23 | 1100.00 | 3.04 | 413.00 | 2.62 | 0.38 | -0.43 |
| 5 | control | 159.30 | 2.20 | 1075.00 | 3.03 | 469.00 | 2.67 | 0.44 | -0.36 |
| 5 | control | 231.80 | 2.37 | 562.00 | 2.75 | 399.00 | 2.60 | 0.71 | -0.15 |
| 5 | control | 150.50 | 2.18 | 814.00 | 2.91 | 405.00 | 2.61 | 0.50 | -0.30 |
| 5 | control | 143.60 | 2.16 | 727.00 | 2.86 | 353.00 | 2.55 | 0.49 | -0.31 |
| 5 | control | 174.20 | 2.24 | 862.00 | 2.94 | 285.00 | 2.45 | 0.33 | -0.48 |
| 5 | control | 151.10 | 2.18 | 765.00 | 2.88 | 370.00 | 2.57 | 0.48 | -0.32 |
| 5 | control | 143.60 | 2.16 | 890.00 | 2.95 | 427.00 | 2.63 | 0.48 | -0.32 |
| 5 | control | 165.50 | 2.22 | 922.00 | 2.96 | 468.00 | 2.67 | 0.51 | -0.29 |
| 5 | control | 119.90 | 2.08 | 917.00 | 2.96 | 454.00 | 2.66 | 0.50 | -0.31 |
| 5 | control | 191.70 | 2.28 | 964.00 | 2.98 | 312.00 | 2.49 | 0.32 | -0.49 |
| 5 | control | 168.50 | 2.23 | 1116.00 | 3.05 | 394.00 | 2.60 | 0.35 | -0.45 |
| 5 | control | 170.70 | 2.23 | 1154.00 | 3.06 | 498.00 | 2.70 | 0.43 | -0.36 |
| 5 | control | 106.10 | 2.03 | 897.00 | 2.95 | 318.00 | 2.50 | 0.35 | -0.45 |
| 5 | control | 178.20 | 2.25 | 1206.00 | 3.08 | 441.00 | 2.64 | 0.37 | -0.44 |
| 5 | control | 155.50 | 2.19 | 805.00 | 2.91 | 350.00 | 2.54 | 0.43 | -0.36 |
| 5 | control | 223.80 | 2.35 | 1323.00 | 3.12 | 315.00 | 2.50 | 0.24 | -0.62 |
| 5 | control | 282.60 | 2.45 | 1417.00 | 3.15 | 411.00 | 2.61 | 0.29 | -0.54 |
| 5 | control | 194.80 | 2.29 | 1316.00 | 3.12 | 383.00 | 2.58 | 0.29 | -0.54 |
| 5 | control | 215.10 | 2.33 | 995.00 | 3.00 | 354.00 | 2.55 | 0.36 | -0.45 |
| 5 | control | 144.80 | 2.16 | 652.00 | 2.81 | 259.00 | 2.41 | 0.40 | -0.40 |
| 5 | control | 152.50 | 2.18 | 1129.00 | 3.05 | 209.00 | 2.32 | 0.19 | -0.73 |
| 5 | control | 149.90 | 2.18 | 1027.00 | 3.01 | 459.00 | 2.66 | 0.45 | -0.35 |
| 5 | control | 245.10 | 2.39 | 1023.00 | 3.01 | 294.00 | 2.47 | 0.29 | -0.54 |
| 6 | control | 186.10 | 2.27 | 961.00 | 2.98 | 243.00 | 2.39 | 0.25 | -0.60 |
| 6 | control | 158.10 | 2.20 | 632.00 | 2.80 | 406.00 | 2.61 | 0.64 | -0.19 |
| 6 | control | 144.40 | 2.16 | 606.00 | 2.78 | 326.00 | 2.51 | 0.54 | -0.27 |
| 6 | control | 175.30 | 2.24 | 1291.00 | 3.11 | 464.00 | 2.67 | 0.36 | -0.44 |
| 6 | control | 126.10 | 2.10 | 1179.00 | 3.07 | 349.00 | 2.54 | 0.30 | -0.53 |
| 6 | control | 124.50 | 2.10 | 1082.00 | 3.03 | 355.00 | 2.55 | 0.33 | -0.48 |
| 6 | control | 130.50 | 2.12 | 1001.00 | 3.00 | 405.00 | 2.61 | 0.40 | -0.39 |
| 6 | control | 184.10 | 2.27 | 1207.00 | 3.08 | 519.00 | 2.72 | 0.43 | -0.37 |
| 6 | control | 169.00 | 2.23 | 1428.00 | 3.15 | 449.00 | 2.65 | 0.31 | -0.50 |
| 6 | control | 189.80 | 2.28 | 1019.00 | 3.01 | 425.00 | 2.63 | 0.42 | -0.38 |
| 6 | control | 183.10 | 2.26 | 1074.00 | 3.03 | 229.00 | 2.36 | 0.21 | -0.67 |
| 6 | control | 243.20 | 2.39 | 1367.00 | 3.14 | 320.00 | 2.51 | 0.23 | -0.63 |
| 6 | control | 167.00 | 2.22 | 1232.00 | 3.09 | 399.00 | 2.60 | 0.32 | -0.49 |
| 6 | control | 155.10 | 2.19 | 929.00 | 2.97 | 289.00 | 2.46 | 0.31 | -0.51 |
| 6 | control | 202.80 | 2.31 | 825.00 | 2.92 | 337.00 | 2.53 | 0.41 | -0.39 |
| 6 | control | 212.70 | 2.33 | 1010.00 | 3.00 | 353.00 | 2.55 | 0.35 | -0.46 |
| 6 | control | 148.30 | 2.17 | 813.00 | 2.91 | 391.00 | 2.59 | 0.48 | -0.32 |
| 6 | control | 218.80 | 2.34 | 858.00 | 2.93 | 379.00 | 2.58 | 0.44 | -0.35 |
| 6 | control | 132.00 | 2.12 | 1250.00 | 3.10 | 337.00 | 2.53 | 0.27 | -0.57 |
| 6 | control | 186.80 | 2.27 | 1064.00 | 3.03 | 372.00 | 2.57 | 0.35 | -0.46 |
| 6 | control | 209.20 | 2.32 | 850.00 | 2.93 | 277.00 | 2.44 | 0.33 | -0.49 |
| 6 | control | 127.60 | 2.11 | 876.00 | 2.94 | 253.00 | 2.40 | 0.29 | -0.54 |
| 6 | control | 167.30 | 2.22 | 627.00 | 2.80 | 291.00 | 2.46 | 0.46 | -0.33 |
| 6 | control | 149.60 | 2.17 | 853.00 | 2.93 | 219.00 | 2.34 | 0.26 | -0.59 |
| 6 | control | 156.10 | 2.19 | 1180.00 | 3.07 | 332.00 | 2.52 | 0.28 | -0.55 |
| 5 | induced | 195.90 | 2.29 | 916.00 | 2.96 | 412.00 | 2.61 | 0.45 | -0.35 |
| 5 | induced | 178.60 | 2.25 | 713.00 | 2.85 | 308.00 | 2.49 | 0.43 | -0.36 |
| 5 | induced | 109.50 | 2.04 | 942.00 | 2.97 | 504.00 | 2.70 | 0.54 | -0.27 |
| 5 | induced | 133.50 | 2.13 | 870.00 | 2.94 | 445.00 | 2.65 | 0.51 | -0.29 |
| 5 | induced | 181.90 | 2.26 | 1051.00 | 3.02 | 491.00 | 2.69 | 0.47 | -0.33 |
| 5 | induced | 158.80 | 2.20 | 1012.00 | 3.01 | 449.00 | 2.65 | 0.44 | -0.35 |
| 5 | induced | 152.30 | 2.18 | 1047.00 | 3.02 | 396.00 | 2.60 | 0.38 | -0.42 |
| 5 | induced | 138.60 | 2.14 | 1071.00 | 3.03 | 397.00 | 2.60 | 0.37 | -0.43 |
| 5 | induced | 205.70 | 2.31 | 1112.00 | 3.05 | 425.00 | 2.63 | 0.38 | -0.42 |
| 5 | induced | 192.60 | 2.28 | 1427.00 | 3.15 | 407.00 | 2.61 | 0.29 | -0.54 |
| 5 | induced | 184.70 | 2.27 | 1559.00 | 3.19 | 514.00 | 2.71 | 0.33 | -0.48 |
| 5 | induced | 161.10 | 2.21 | 784.00 | 2.89 | 341.00 | 2.53 | 0.43 | -0.36 |
| 5 | induced | 231.30 | 2.36 | 693.00 | 2.84 | 352.00 | 2.55 | 0.51 | -0.29 |
| 5 | induced | 186.60 | 2.27 | 743.00 | 2.87 | 359.00 | 2.56 | 0.48 | -0.32 |
| 5 | induced | 139.30 | 2.14 | 756.00 | 2.88 | 365.00 | 2.56 | 0.48 | -0.32 |
| 5 | induced | 181.10 | 2.26 | 819.00 | 2.91 | 395.00 | 2.60 | 0.48 | -0.32 |
| 5 | induced | 164.10 | 2.22 | 1072.00 | 3.03 | 379.00 | 2.58 | 0.35 | -0.45 |
| 5 | induced | 196.50 | 2.29 | 857.00 | 2.93 | 374.00 | 2.57 | 0.44 | -0.36 |
| 5 | induced | 193.00 | 2.29 | 1227.00 | 3.09 | 333.00 | 2.52 | 0.27 | -0.57 |
| 5 | induced | 206.70 | 2.32 | 1097.00 | 3.04 | 305.00 | 2.48 | 0.28 | -0.56 |
| 5 | induced | 182.60 | 2.26 | 783.00 | 2.89 | 261.00 | 2.42 | 0.33 | -0.48 |
| 6 | induced | 227.30 | 2.36 | 1322.00 | 3.12 | 572.00 | 2.76 | 0.43 | -0.36 |
| 6 | induced | 221.60 | 2.35 | 1342.00 | 3.13 | 428.00 | 2.63 | 0.32 | -0.50 |
| 6 | induced | 238.00 | 2.38 | 928.00 | 2.97 | 422.00 | 2.63 | 0.45 | -0.34 |
| 6 | induced | 172.30 | 2.24 | 953.00 | 2.98 | 618.00 | 2.79 | 0.65 | -0.19 |
| 6 | induced | 216.20 | 2.33 | 1327.00 | 3.12 | 437.00 | 2.64 | 0.33 | -0.48 |
| 6 | induced | 209.10 | 2.32 | 1090.00 | 3.04 | 391.00 | 2.59 | 0.36 | -0.45 |
| 6 | induced | 214.60 | 2.33 | 651.00 | 2.81 | 473.00 | 2.67 | 0.73 | -0.14 |
| 6 | induced | 254.30 | 2.41 | 745.00 | 2.87 | 466.00 | 2.67 | 0.63 | -0.20 |
| 6 | induced | 228.70 | 2.36 | 827.00 | 2.92 | 517.00 | 2.71 | 0.63 | -0.20 |
| 6 | induced | 189.90 | 2.28 | 967.00 | 2.99 | 739.00 | 2.87 | 0.76 | -0.12 |
| 6 | induced | 195.00 | 2.29 | 612.00 | 2.79 | 454.00 | 2.66 | 0.74 | -0.13 |
| 6 | induced | 151.40 | 2.18 | 849.00 | 2.93 | 445.00 | 2.65 | 0.52 | -0.28 |
| 6 | induced | 214.20 | 2.33 | 894.00 | 2.95 | 439.00 | 2.64 | 0.49 | -0.31 |
| 6 | induced | 204.90 | 2.31 | 1005.00 | 3.00 | 364.00 | 2.56 | 0.36 | -0.44 |
| 6 | induced | 162.50 | 2.21 | 794.00 | 2.90 | 377.00 | 2.58 | 0.47 | -0.32 |
| 6 | induced | 226.50 | 2.36 | 924.00 | 2.97 | 343.00 | 2.54 | 0.37 | -0.43 |
| 6 | induced | 199.80 | 2.30 | 917.00 | 2.96 | 344.00 | 2.54 | 0.38 | -0.43 |
| 6 | induced | 224.10 | 2.35 | 1015.00 | 3.01 | 328.00 | 2.52 | 0.32 | -0.49 |
| 6 | induced | 220.40 | 2.34 | 974.00 | 2.99 | 376.00 | 2.58 | 0.39 | -0.41 |
| 6 | induced | 161.50 | 2.21 | 767.00 | 2.88 | 444.00 | 2.65 | 0.58 | -0.24 |
| 6 | induced | 205.30 | 2.31 | 957.00 | 2.98 | 300.00 | 2.48 | 0.31 | -0.50 |

Table S8: Data associated with figure 2B.

| instar | treatment | volumetric measurements (µm^3) | log (volume) |
| --- | --- | --- | --- |
| 3 | induced | 9213406652.00 | 9.96 |
| 3 | control | 1362511634.00 | 9.13 |
| 3 | induced | 1506226896.00 | 9.18 |
| 3 | induced | 1556128724.00 | 9.19 |
| 3 | control | 1893818657.00 | 9.28 |
| 3 | induced | 1995861568.00 | 9.30 |
| 3 | induced | 2001650949.00 | 9.30 |
| 3 | control | 2028903392.00 | 9.31 |
| 3 | control | 2030476404.00 | 9.31 |
| 3 | induced | 2043972536.00 | 9.31 |
| 3 | control | 2056053807.00 | 9.31 |
| 3 | control | 2135630125.00 | 9.33 |
| 3 | control | 216876882.00 | 8.34 |
| 3 | control | 2229646782.00 | 9.35 |
| 3 | induced | 2247772145.00 | 9.35 |
| 3 | induced | 2340143802.00 | 9.37 |
| 3 | induced | 2378246453.00 | 9.38 |
| 3 | control | 2378767637.00 | 9.38 |
| 3 | control | 2381906491.00 | 9.38 |
| 3 | induced | 2688631178.00 | 9.43 |
| 3 | control | 2744294804.00 | 9.44 |
| 3 | induced | 3049043901.00 | 9.48 |
| 3 | control | 3195531821.00 | 9.50 |
| 3 | control | 3377205768.00 | 9.53 |
| 3 | control | 3421392389.00 | 9.53 |
| 3 | induced | 3645196944.00 | 9.56 |
| 3 | control | 3997396583.00 | 9.60 |
| 3 | induced | 4128627841.00 | 9.62 |
| 3 | induced | 4164475399.00 | 9.62 |
| 4 | control | 1307434876.00 | 9.12 |
| 4 | control | 1479002866.00 | 9.17 |
| 4 | control | 1810481653.00 | 9.26 |
| 4 | control | 1883317813.00 | 9.27 |
| 4 | control | 2061482937.00 | 9.31 |
| 4 | induced | 2091581905.00 | 9.32 |
| 4 | induced | 2203315001.00 | 9.34 |
| 4 | control | 2233856295.00 | 9.35 |
| 4 | control | 2829906638.00 | 9.45 |
| 4 | control | 309145731.00 | 8.49 |
| 4 | control | 321563103.00 | 8.51 |
| 4 | induced | 3313287324.00 | 9.52 |
| 4 | control | 3475363858.00 | 9.54 |
| 4 | induced | 3653835394.00 | 9.56 |
| 4 | control | 3667674246.00 | 9.56 |
| 4 | induced | 3687801623.00 | 9.57 |
| 4 | induced | 3782146321.00 | 9.58 |
| 4 | induced | 3935746557.00 | 9.60 |
| 4 | control | 3936938589.00 | 9.60 |
| 4 | induced | 4517572233.00 | 9.65 |
| 4 | control | 4768509691.00 | 9.68 |
| 4 | induced | 4813988772.00 | 9.68 |
| 4 | control | 4949947481.00 | 9.69 |
| 4 | induced | 530243992.00 | 8.72 |
| 4 | induced | 5580264354.00 | 9.75 |
| 4 | induced | 5589609713.00 | 9.75 |
| 4 | induced | 5639234763.00 | 9.75 |
| 4 | control | 5890510583.00 | 9.77 |
| 4 | induced | 6818884176.00 | 9.83 |
| 4 | induced | 6834502452.00 | 9.83 |
| 5 | control | 1826282219.00 | 9.26 |
| 5 | control | 186962218.00 | 8.27 |
| 5 | control | 1917060114.00 | 9.28 |
| 5 | control | 2103487426.00 | 9.32 |
| 5 | control | 2124661988.00 | 9.33 |
| 5 | induced | 2291754019.00 | 9.36 |
| 5 | control | 2321807265.00 | 9.37 |
| 5 | control | 2336126847.00 | 9.37 |
| 5 | induced | 2347619813.00 | 9.37 |
| 5 | induced | 240016.80 | 5.38 |
| 5 | control | 2516764945.00 | 9.40 |
| 5 | control | 2718449258.00 | 9.43 |
| 5 | induced | 2765704941.00 | 9.44 |
| 5 | control | 304395203.00 | 8.48 |
| 5 | control | 3175498057.00 | 9.50 |
| 5 | control | 3222701448.00 | 9.51 |
| 5 | induced | 3476145653.00 | 9.54 |
| 5 | control | 385662323.00 | 8.59 |
| 5 | control | 3993528598.00 | 9.60 |
| 5 | control | 4055178471.00 | 9.61 |
| 5 | control | 4328477683.00 | 9.64 |
| 5 | induced | 436259017.00 | 8.64 |
| 5 | control | 4519685836.00 | 9.66 |
| 5 | control | 4716585477.00 | 9.67 |
| 5 | control | 4928387246.00 | 9.69 |
| 5 | induced | 5157758038.00 | 9.71 |
| 5 | induced | 5267821663.00 | 9.72 |
| 5 | control | 5518596659.00 | 9.74 |
| 5 | control | 5844273028.00 | 9.77 |
| 5 | induced | 6149071502.00 | 9.79 |
| 5 | induced | 6534225621.00 | 9.82 |
| 5 | control | 674388857.00 | 8.83 |
| 5 | induced | 9177378289.00 | 9.96 |
| 5 | induced | 1076745178.00 | 9.03 |
| 5 | induced | 1283557837.00 | 9.11 |

Table S9: Data associated with figure 2B.1, B.2, B.3.

| instar | treatment | volume optic ganglia µm^3 | log(volume OG) |
| --- | --- | --- | --- |
| 3 | control | 72670.96 | 4.86 |
| 3 | control | 86042.79 | 4.93 |
| 3 | control | 86273.81 | 4.94 |
| 3 | control | 93574.60 | 4.97 |
| 3 | control | 40626.62 | 4.61 |
| 3 | induced | 81246.36 | 4.91 |
| 3 | induced | 65596.55 | 4.82 |
| 3 | induced | 82675.15 | 4.92 |
| 3 | induced | 99709.68 | 5.00 |
| 3 | induced | 119689.06 | 5.08 |
| 4 | control | 88358.62 | 4.95 |
| 4 | control | 57444.20 | 4.76 |
| 4 | control | 227801.35 | 5.36 |
| 4 | control | 188973.49 | 5.28 |
| 4 | control | 161493.18 | 5.21 |
| 4 | induced | 134659.03 | 5.13 |
| 4 | induced | 120021.19 | 5.08 |
| 4 | induced | 203172.98 | 5.31 |
| 4 | induced | 240089.58 | 5.38 |
| 4 | induced | 118897.72 | 5.08 |
| 4 | induced | 170282.96 | 5.23 |
| 4 | induced | 199626.86 | 5.30 |
| 5 | control | 124881.17 | 5.10 |
| 5 | control | 213467.46 | 5.33 |
| 5 | control | 252457.46 | 5.40 |
| 5 | control | 189255.27 | 5.28 |
| 5 | control | 283433.91 | 5.45 |
| 5 | control | 293652.08 | 5.47 |
| 5 | induced | 160386.60 | 5.21 |
| 5 | induced | 230919.25 | 5.36 |
| 5 | induced | 231240.44 | 5.36 |
| 5 | induced | 295934.93 | 5.47 |
| 5 | induced | 377788.43 | 5.58 |

Table S10: Data associated with figure 3B.

| instar | treatment | volumetric measurements µm^3 |
| --- | --- | --- |
| 5 | control | 350554.4537 |
| 5 | control | 486951.2549 |
| 5 | control | 241214.9684 |
| 5 | control | 246528.4077 |
| 5 | control | 367065.8282 |
| 5 | induced | 264457.8016 |
| 5 | induced | 259315.5072 |
| 5 | induced | 226520.3527 |
| 5 | induced | 493099.0003 |
| 6 | control | 408472.7806 |
| 6 | control | 417457.266 |
| 6 | control | 321402.897 |
| 6 | control | 421329.6636 |
| 6 | induced | 517929.1201 |
| 6 | induced | 420838.6379 |
| 6 | control | 319621.9522 |
| 6 | control | 213457.7694 |
| 6 | induced | 375948.4724 |
| 6 | induced | 425371.2689 |
| 6 | induced | 386035.2066 |
| 6 | induced | 437051.961 |
| 6 | induced | 428243.5768 |
| 6 | induced | 401654.9729 |
